# Morpholine-based buffers activate aerobic photobiocatalysis *via* spin correlated ion pair formation†
†Electronic supplementary information (ESI) available: Experimental conditions, supplementary figures and references. See DOI: 10.1039/c8cy02524j


**DOI:** 10.1039/c8cy02524j

**Published:** 2019-02-11

**Authors:** Leticia C. P. Gonçalves, Hamid R. Mansouri, Erick L. Bastos, Mohamed Abdellah, Bruna S. Fadiga, Jacinto Sá, Florian Rudroff, Marko D. Mihovilovic

**Affiliations:** a Institute of Applied Synthetic Chemistry , TU Wien , Getreidemarkt 9/163 , 1060 Vienna , Austria . Email: leticia.goncalves@tuwien.ac.at; b Department of Fundamental Chemistry , Institute of Chemistry , University of São Paulo , 03178-200 São Paulo , Brazil; c Physical Chemistry Division , Department of Chemistry , Ångström Laboratory , Uppsala University , 75120 Uppsala , Sweden; d Department of Chemistry , Qena Faculty of Science , South Valley University , 83523 Qena , Egypt; e Institute of Physical Chemistry , Polish Academy of Sciences , 01-224 Warsaw , Poland

## Abstract

The use of enzymes for synthetic applications is a powerful and environmentally-benign approach to increase molecular complexity. Oxidoreductases selectively introduce oxygen and hydrogen atoms into myriad substrates, catalyzing the synthesis of chemical and pharmaceutical building blocks for chemical production. However, broader application of this class of enzymes is limited by the requirements of expensive cofactors and low operational stability. Herein, we show that morpholine-based buffers, especially 3-(*N*-morpholino)propanesulfonic acid (MOPS), promote photoinduced flavoenzyme-catalyzed asymmetric redox transformations by regenerating the flavin cofactor *via* sacrificial electron donation and by increasing the operational stability of flavin-dependent oxidoreductases. The stabilization of the active forms of flavin by MOPS *via* formation of the spin correlated ion pair ^3^[flavin˙^–^–MOPS˙^+^] ensemble reduces the formation of hydrogen peroxide, circumventing the oxygen dilemma under aerobic conditions detrimental to fragile enzymes.

## Abstract 2

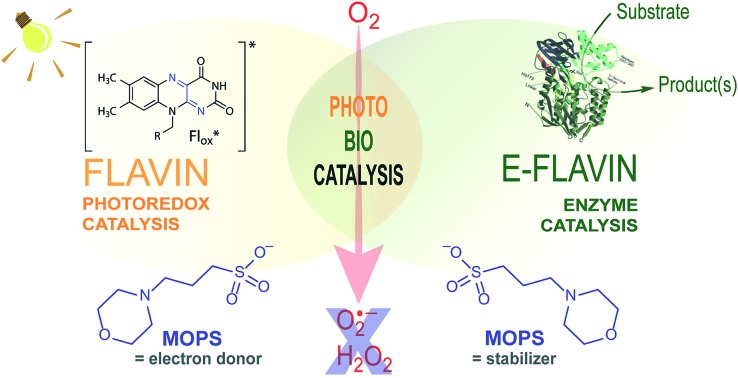
MOPS acts as a Good buffer and electron donor and prevents the degradation of catalysts by reactive species in aerated photobiocatalysis.

## Introduction

**Fig. 1:**
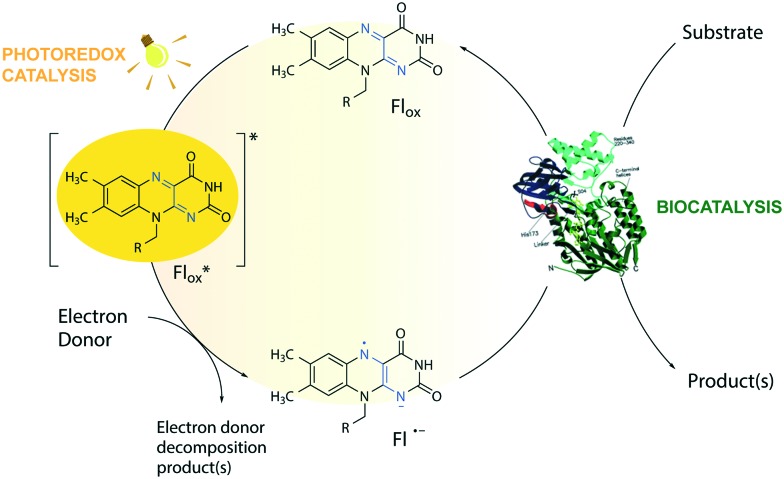
Flavin-mediated photoredox and enzyme catalysis. Reduction of flavins (Fl) occurs upon light irradiation in the presence of electron donors (EDs). The excited flavin (Fl*ox) oxidizes an ED resulting in a semiquinone radical anion (Fl˙^–^), which mediates the transfer of reducing equivalents to the active sites of flavin-dependent enzymes. For simplicity, protonation equilibria are not shown. R = flavin adenine dinucleotide (FAD) or flavin mononucleotide (FMN).

The toolbox of synthetic chemists includes photoredox and biocatalytic transformations that benefit from the use of light energy.^1^,^2^ Flavin-dependent oxidoreductases are important biocatalysts for various industrial applications^3^ but require the reduction of their bound flavin cofactor, typically flavin adenine dinucleotide (FAD) or flavin mononucleotide (FMN), to complete their catalytic cycle. Photoexcitation in the presence of sacrificial electron donors (EDs, Fig. 1) is a convenient method to reduce and thereby regenerate the flavin cofactor. This approach circumvents the need for expensive additional cofactors such as dihydronicotinamide adenine dinucleotide phosphate (NADPH).^1^ Low cost amines, such as ethylenediaminetetraacetic acid (EDTA) and triethanolamine (TEOA), are well-known sacrificial EDs in photoredox catalysis and able to reduce photoexcited flavins.^1^,^4^

The limited operational stability of flavin-dependent oxidoreductases compromises their broad application, especially under cell-free conditions.^5^ During the photoredox cycle, electron and energy transfer reactions between excited flavin and molecular oxygen produce deleterious reactive oxygen species (ROS) affecting the integrity of the enzyme.^6^–^8^ Photoinduced enzyme-catalyzed reactions, or photobiocatalysis, under anaerobic conditions do not face this so-called ‘*oxygen dilemma*’.^1^,^9^,^10^ However, this approach is not applicable to systems that employ enzymes that require oxygen for catalysis such as flavin-containing monooxygenases (FMOs). Attempts to reduce the formation of ROS by replacing flavin with less oxidizable deazaflavins were not successful.^11^ Stabilization of the triplet state of the excited flavin (Fl_ox_) and the reactive semiquinone radical anion (Fl˙^–^) is expected to improve the efficiency and applicability of light-driven enzymatic reactions by avoiding the formation of ROS side products *via* energy or electron transfer in the presence of oxygen. However, no additive has been shown to affect the reaction by this mechanism, so far.

MOPS (3-(*N*-morpholino)propanesulfonic acid) and MES (3-(*N*-morpholino)ethanesulfonic acid) are Good's buffers^12^ used in biology because of their chemical inertness.^13^ The photoreduction of riboflavin and deazaflavin in the presence of a morpholine buffer, however, suggests that the buffer may act as a sacrificial ED.^11^,^14^ Herein, we show that MOPS not only acts as a sacrificial ED to regenerate the flavin cofactor in flavin oxidoreductase photobiocatalysis, but also stabilizes the triplet state of the excited flavin and the reactive flavin semiquinone radical anion preventing to some extent the inactivation of fragile flavin-dependent oxidoreductases by ROS under aerobic conditions, contributing to circumvention of the *oxygen dilemma* in aerated photobiocatalysis and expanding its applicability to enzymes that require oxygen for catalysis.

## Experimental

### Standard photoredox enzyme-catalysed reactions

Photoredox biotransformations were performed in a 96-well plate sealed with transparent sealing films, unless otherwise stated. Ketoisophorone (**1a**) reduction (1 mM) was performed in the presence of FMN (100 μM final concentration) employing XenB (10 μM) as a reductase. The Baeyer–Villiger oxidation of cyclohexanone (**2a**), 2-phenylcyclohexanone (**3a**) or bicyclo[3.2.0]hept-2-en-6-one (**4a**) (1 mM final concentration) was performed in the presence of FAD (100 μM final concentration) and NADP^+^ (0.25 mM) employing CHMO_Acineto_ (10 μM) as a biocatalyst. MOPS buffer (100 mM, pH 7.5) was employed as an electron donor and medium, unless otherwise stated. Control reactions were performed in 100 mM Tris-HCl (pH 7.5) in the presence or absence of 25 mM EDTA and in 100 mM sodium phosphate buffer (pH 7.5). The plate was placed over a cooling block in order to keep the reactions at 30 ± 3 °C. The reactants in the plate were irradiated for a certain time using a 300 W daylight lamp (Ultra Vitalux, Osram) without a filter. Screening samples were taken at *t* = 0 h (immediately after mixing) and after a certain time of incubation. Reference reactions were performed with the same biocatalysts using the enzymatic recycling system GDH (5 U mL^–1^)/glucose (12 mM) in the presence of NADP^+^ (0.25 mM). Control experiments were performed in the dark or in the absence of the biocatalyst.

### Nanosecond laser flash spectroscopy

Nanosecond transient absorption kinetics were recorded using a frequency tripled Q-switched Nd:YAG laser coupled with an OPO (Quantel Brilliant B) to obtain the desired wavelength for the pump light (5 ns pulse at 10 Hz). A detection system from Applied Photophysics (LKS80 spectrometer) was used, equipped with a Xe arc lamp (pulsed or continuous wave), two monochromators and an R928-type PMT read using a 600 MHz oscilloscope (Agilent Technologies Infinium 10 GSa s^–1^) for kinetics. The kinetics were collected with the maximum resolution producing 20 000 point traces that were logarithmically oversampled starting from 50 points from the excitation about 10 ns after the pulse. The excitation light wavelength was 445 nm. The excitation light power was controlled by means of neutral density filters. Experiments were carried out in a quartz cuvette employing 100 μM FAD in 100 mM MOPS at pH 7.5.

### Steady-state absorption measurements

The experiments were performed in a quartz cuvette using the following conditions unless otherwise stated: 100 μM FAD in 100 mM MOPS or Tris-HCl (pH 7.5) in the presence or absence of EDTA (25 mM). The cuvette cell was mounted in a support with ports for the UV-vis fibre optic probe from Ocean Optics capable of collecting a full UV-vis spectrum in less than 10 s. The experiments consisted of sample illumination for 60 min with a 445 nm CW laser, followed by a 30 min dark period.

## Results and discussion

### Proof-of-concept

In order to develop a simple and versatile light-induced regeneration system for both oxygen dependent and independent flavin-containing enzymes, we chose two oxidoreductases as model biocatalysts: first, XenB (an enoate reductase from *Pseudomonas* sp.)^15^ for the oxygen-independent enantioselective reduction of ketoisophorone (**1a**) to levodione (**1b**), and second, cyclohexanone monooxygenase from *Acinetobacter calcoaceticus* NCIMB 9871 (CHMO_Acineto_), a well-known Baeyer–Villiger monooxygenase (BVMO) for the oxidation of cyclohexanone (**2a**) to ε-caprolactone (**2b**) under aerated conditions.^16^ Upon daylight irradiation, the effect of morpholine-based buffers on the photobiocatalytic process involving XenB and CHMO_Acineto_ was evaluated under aerobic conditions. EDTA in Tris-HCl buffer was used as a positive control.^17^,^18^ Sodium phosphate buffer (NaPi) in the absence of an ED was used as the negative control. The flavin prosthetic group of each enzyme was added to the reaction as a photosensitizer in sub-stoichiometric quantities (*i.e.* FMN was added to XenB and FAD to CHMO_Acineto_). The nicotinamide cofactor NADP^+^ was added to all the reactions with CHMO_Acineto_ due to its involvement in the mechanism of the catalysis of this enzyme^19^ but not to the reactions with XenB.

**Fig. 2:**
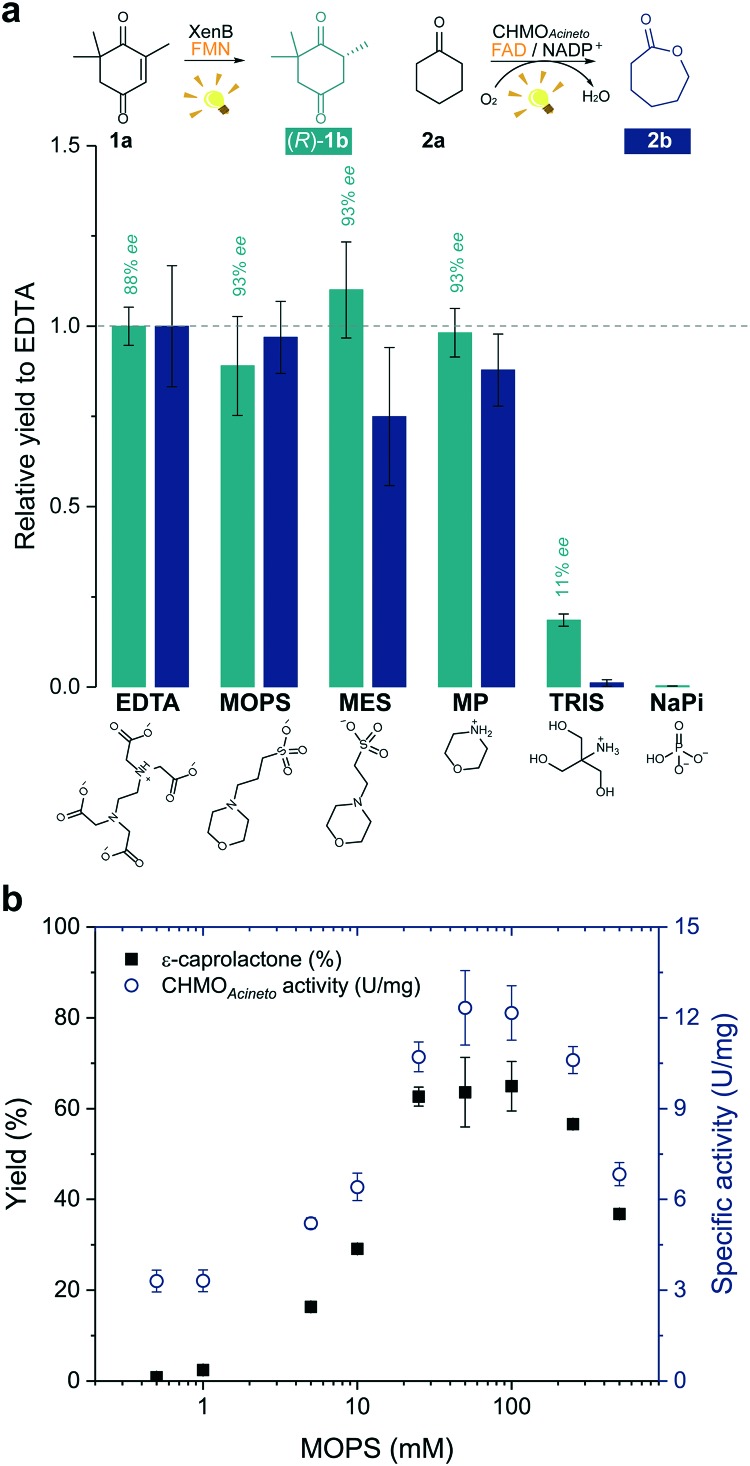
Morpholine-based buffers and solutions promote photoinduced enzyme catalysis. (a) Effect of the electron donor or buffer (EDTA/Tris-HCl, MOPS, MES, morpholine (MP), Tris-HCl and sodium phosphate (NaPi)) on the photoinduced biotransformations of ketoisophorone (**1a**) and cyclohexanone (**2a**) after 1 h irradiation. Product yields were normalized by the yield obtained on the reaction with EDTA (dashed line). Structures below the graph represent the main species at pH 7.5. (b) Effect of the concentration of MOPS on the ε-caprolactone yield (**2b**) and on the specific enzyme activity (U mg^–1^). Reactions involving CHMO_Acineto_ was supplied with NADP^+^ (see experimental details in the Experimental section). NADPH was not added to any reaction. Level of statistical significance, *p* < 0.05.

After 1 h of light irradiation, **1b** and **2b** were formed in MOPS and MES buffers and in the non-buffered morpholine (MP) solution (Fig. 2a) in the absence of NADPH. Gas chromatography (GC) yields relative to the EDTA/Tris-HCl conditions are statistically comparable to those of the positive control except in the case of **2b** in MES. Identical selectivity has been achieved under all conditions. Nevertheless, the photoreduction of **1a** to (*rac*)-**1b** was observed in Tris-HCl buffer in the absence of EDTA as an ED but in poor yields (Fig. 2 and Fig. S1†), and no product was detected in control experiments with MOPS in the dark (Fig. S1 and S2†).

The enzyme-catalysed Baeyer–Villiger oxidation of **2a** with CHMO_Acineto_/FAD/NADP^+^ was defined as the study system since it requires oxygen for catalysis. MOPS (p*K*_a_ 7.15)^20^ assures full buffering capacity at pH 7.5, an adequate operational pH value for several enzymes, which does not match with MES (p*K*_a_ 6.1).^20^ During the optimization of reaction conditions, we found that both the product yield and enzyme stability depend on the concentration of MOPS (0–500 mM) (Fig. 2b). At low concentrations of MOPS (<1 mM), hardly any conversion to the desired lactone **2b** was observed after 1 h irradiation, probably due to inactivation or degradation of the enzyme and the photocatalyst FAD. Yields higher than 60% were obtained above 25 mM MOPS, with the maximum yield reached at 50–100 mM MOPS; further increase in MOPS concentration decreased the reaction yield. Intrigued by these results, we investigated the specific enzyme activity of CHMO_Acineto_ at different MOPS concentrations (Fig. 2b). The enzyme activity profile is similar to the one observed for the conversion of **2a** and drops 2-fold as the MOPS concentration increases above 100 mM. Although 50 mM MOPS was enough to reach the maximum yield, we defined 100 mM as the standard concentration of MOPS to assure its function as both a buffer and ED.

### Reaction performance and selectivity

**Fig. 3:**
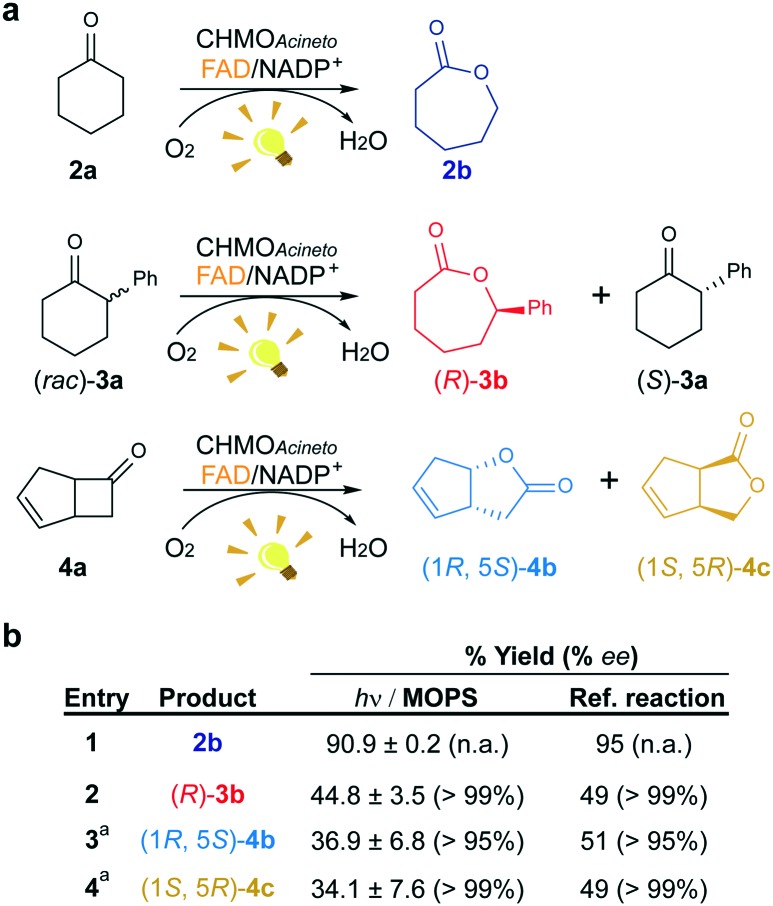
Maintenance of the stereo- and regioselectivity of CHMO_Acineto_. (a) Substrates employed in the photoinduced biotransformations with CHMO_Acineto_ in the presence of FAD and NADP^+^ in MOPS (100 mM, pH 7.5). (b) Maximum yield (%) and enantiomeric excess (% ee) determined by GC analysis. Reference reaction was performed using the enzymatic recycling system GDH/glucose/NADP^+^. ^a^Results are shown in terms of conversion for this substrate. n.a.: not applicable. Control experiments are shown in Fig. S7.†

We investigated the effect of the concentration of CHMO_Acineto_, the flavin cofactor (FAD) and the nicotinamide cofactor (NADP^+^) on the oxidation of **2a** (1 mM) to gain deeper mechanistic insights. Reaction yields depend on the concentration of the enzyme and FAD (Fig. S3a and S3b†) but are independent of the concentration of NADP^+^ (>50 μM, Fig. S3c†). Although NADP^+^ is associated with the stabilization of the flavin peroxide intermediate necessary for catalysis by BVMOs,^19^ this cofactor is not necessary for the photoredox cycle. Under standard work conditions (10 μM CHMO_Acineto_, 100 μM FAD and 250 μM NADP^+^), 90% yield (full conversion) was achieved after 6 h of irradiation (TOF = 32 h^–1^, Fig. 3 and S4†).

To confirm the maintenance of the stereo- and regioselectivity of CHMO_Acineto_ in the photoinduced process mediated by MOPS as an ED, two other substrates were tested: 2-phenylcyclohexanone (**3a**) and bicyclo[3.2.0]hept-2-en-6-one (**4a**) (Fig. 3a). The enzyme selectivity analysis with **3a** (Fig. S5†) and **4a** (Fig. S6†) shows that the typical stereo- and regioselectivities of CHMO_Acineto_ are maintained (Fig. 3b). The maximum yield and enantiomeric excess are comparable to the results obtained employing an enzymatic recycling system (GDH/Glu/NADP^+^) as the source of reducing equivalents in the dark (Fig. 3b).

### Mechanism of catalysis enhanced by MOPS

We determined the stability of the CHMO_Acineto_/FAD system in MOPS and Tris-HCl buffers (100 mM, pH 7.5) at 30 °C (Fig. S8†) in the dark to assure that MOPS does not deactivate our model enzyme. The half-life of CHMO_Acineto_ showed a 2-fold increase in MOPS (*t*_1/2_ = 25.4 h) in comparison with that of Tris-HCl (*t*_1/2_ = 12.4 h) under the same conditions and a *ca.* 14-fold increase compared to that obtained in 50 mM Tris at pH 8.5 using an enzyme sample purified in the absence of FAD (*t*_1/2_ = 1.87 h).^6^ Nevertheless, the enzyme activity in MOPS remained at 60% of its initial value whereas it reached 20% in Tris-HCl after 50 h of incubation (Fig. S8†), indicating that MOPS protects the enzyme from deactivation.

The photodegradation of flavins is a known obstacle to the application of flavin derivatives as photosensitizers.^21^ ROS can occur *via* the diffusion-controlled electron transfer from photoreduced flavins to oxygen, energy transfer from ^3^FAD* to ^3^O_2_ and/or direct decomposition of flavin hydroperoxide.^6^,^7^ We found that the concentration of H_2_O_2_ produced by irradiation of FAD in Tris-HCl buffer for 4 h is four-times larger than that measured in MOPS buffer, *i.e.* 118 μM (initial rate: 36 μM h^–1^) *vs.* 28 μM. Yet, a negligible amount of H_2_O_2_ was determined before 4 h in the system FAD/MOPS (Fig. S9†). These results show that the MOPS buffer does not only act as an electron donor but also protects fragile flavin-dependent enzymes and the flavin cofactor from the oxidative deactivation inherent to aerobic conditions.

**Fig. 4:**
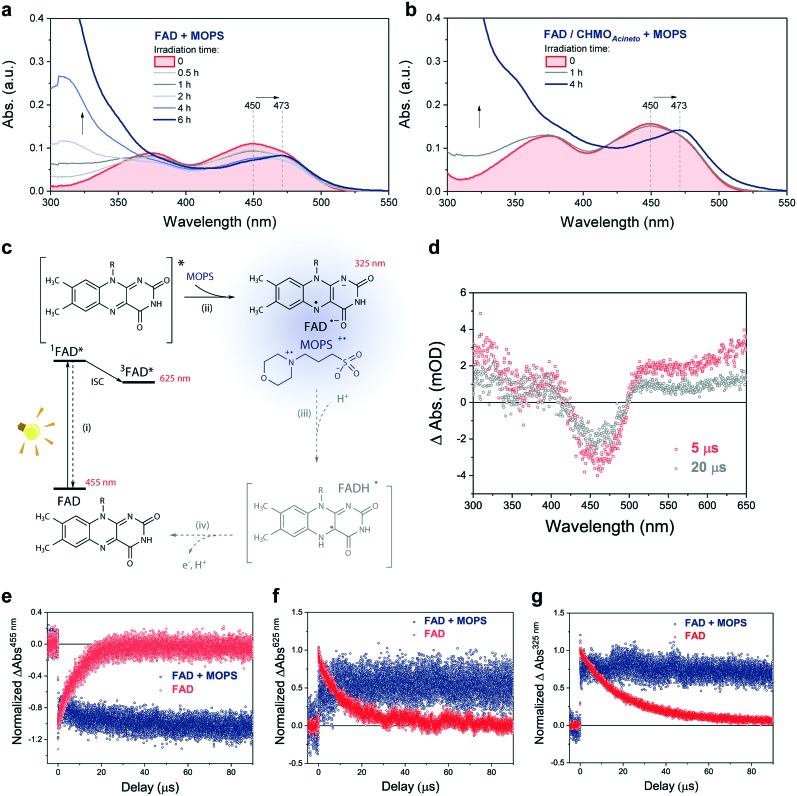
MOPS prolongs the lifetime of the excited FAD species. Effect of MOPS on the absorption spectra of FAD over the time of irradiation in the (a) absence and (b) presence of CHMO_Acineto_/**2a**/NADP^+^. (c) Proposed species formed upon excitation of FAD in the presence of MOPS. (d) Time-resolved light-induced absorption changes for FAD in water at 5 and 20 μs after light excitation (*λ*_EX_ = 445 nm) under argon. Effect of MOPS on the kinetic traces extracted at (e) 455 nm (FAD ground state absorption), (f) 655 nm (FAD triplet state absorption), and (g) 325 nm (FAD anionic semiquinone).

The photochemical reaction between MOPS and other EDs with FAD was studied in the presence and absence of the system CHMO_Acineto_/**1a** by using steady-state absorption spectroscopy (Fig. S11†). In the absence of the enzyme, MOPS drastically reduces the rate of disappearance of FAD, monitored at 450 nm over a period of 6 h, but causes an increase in near-UV absorption and at 473 nm, which has been associated with the formation of the anionic form of the flavin semiquinone, FAD˙^–^ (Fig. 4a–c).^22^,^23^ Subsequent reduction of oxygen producing superoxide would be prevented if the radical anion semiquinone is stabilized by the formation of spin-correlated radical–ion pairs ^1^[FAD˙^(↑)–^–MOPS˙^(↓)+^] and ^3^[FAD˙^(↑)–^–MOPS˙^(↑)+^]. The presence of CHMO_Acineto_*/***1a** does not affect the absorption profiles dramatically (Fig. 4b). However, the decrease in absorption at 450 nm over a period of 1 h is less pronounced in all cases due to the fast re-oxidation of the reduced flavin under turnover conditions.

Light-induced changes in the time-resolved absorption spectrum of FAD 5 μs and 20 μs after excitation (*λ*_EX_ = 445 nm) were studied in the presence and absence of MOPS to rationalize the role of this ED on the dynamics of FAD depletion (Fig. 4d). The control experiment in the absence of MOPS and under an argon atmosphere shows bleaching of the peak at 455 nm (FAD) and two transient increases of absorption in the green/red and near-UV regions, as reported previously.^24^ Kinetic tracing showed that the bleach signal associated with the FAD ground state recovers almost completely within 30 μs (Fig. 4e). The signal in the green/red region corresponds to the absorption of ^3^FAD*, and its depletion (Fig. 4f) is related to the formation of FAD˙^–^, inferred from the signal in the near-UV region.^25^,^26^ In the absence of MOPS, FAD˙^–^ decays almost completely within 50–60 μs, (Fig. 4g). The addition of MOPS did not change the spectral profile of FAD but has a major kinetic effect (Fig. 4e–g). The intensity of the signals related to ^3^FAD* and FAD˙^–^, monitored at 625 nm and 325 nm, respectively, becomes practically constant within 100 μs, indicating the stabilization of the anionic semiquinone, possibly *via* the ^3^[FAD˙^–^–MOPS˙^+^] ensemble, and resulting in effective ‘freezing’ of photoinduced states (Fig. 4c). The ^3^[FAD˙^–^–MOPS˙^+^] ensemble can *a priori* undergo electron back transfer to produce ^3^FAD* and MOPS if the Gibbs energy of annihilation is higher than the triplet energy of ^3^FAD*.^27^ The non-recovery of the ground state is archetypal of excited state stabilization, despite the fact that such strong stabilization is often associated with irreversible processes. Experiments in aerated solution did not differ significantly from experiments under an argon atmosphere when MOPS was present (Fig. S12†). Experiments containing only the enzyme are not feasible due to enzyme instability in the absence of its excess cofactor FAD, which is not tightly bound to our model enzyme CHMO_Acineto_.^6^

**Fig. 5:**
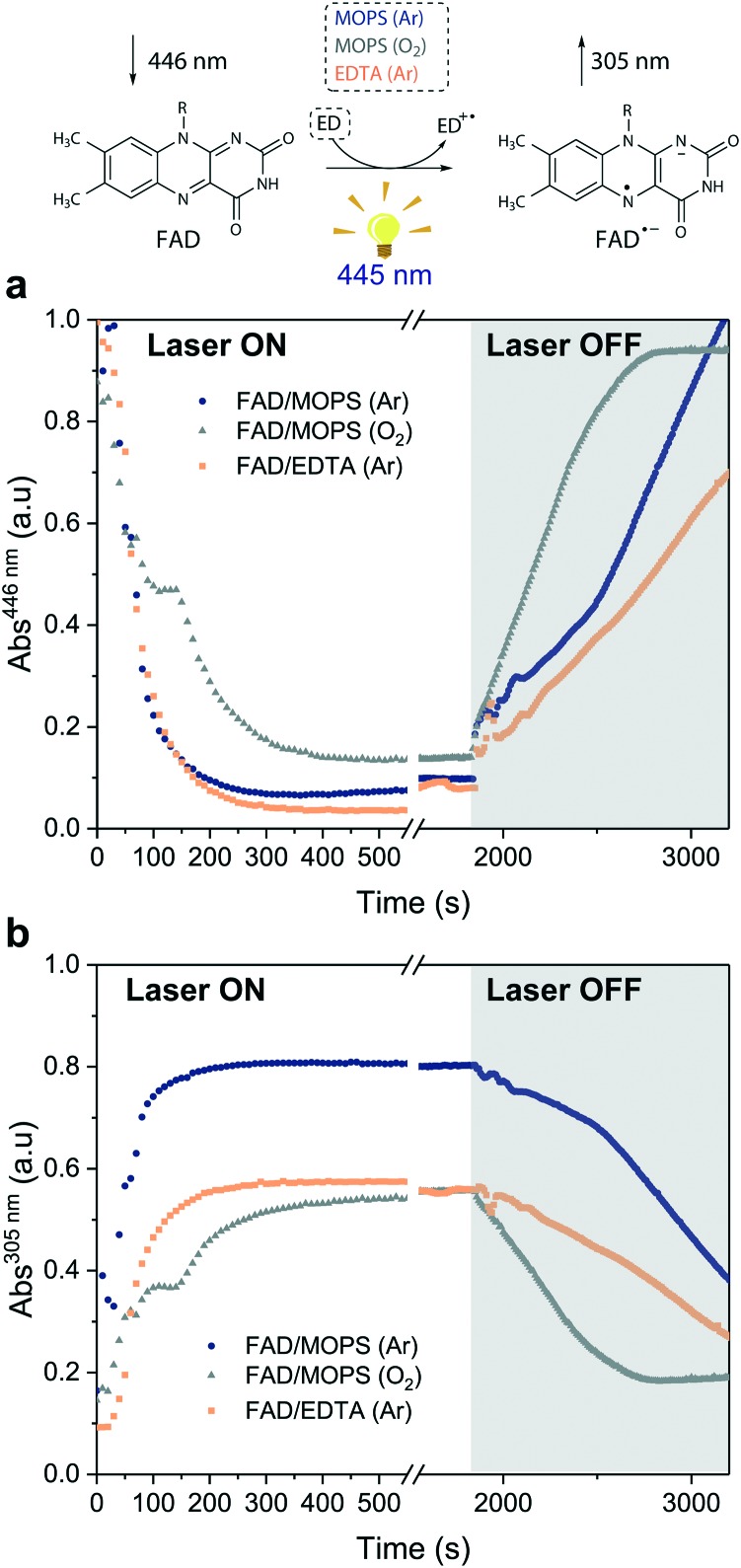
Steady-state absorption measurements under light excitation (*λ*_EX_ = 445 nm). Kinetic traces extracted from the absorption spectra of the reactions between: (i) FAD and MOPS in the absence (dark blue circles) and in the presence of oxygen (gray triangles) and (ii) FAD and EDTA (orange squares) in Tris-HCl. Kinetic traces at (a) 446 nm (FAD ground-state) and (b) 305 nm (FAD anionic semiquinone). The laser was switched off after 30 min of irradiation (gray region).

Excitation of FAD with a 445 nm laser in the presence of MOPS and under an argon atmosphere resulted in simultaneous fast bleaching of ground-state absorption (Fig. 5a) and formation of a signal at 305 nm (Fig. 5b) that was not observed in control experiments in the absence of either FAD or MOPS (Fig. S13†). The rate of formation and steady-state concentration of the species absorbing at 305 nm decrease with decreasing MOPS concentration (Fig. S14a†). Therefore, it is reasonable to infer that the signal at 305 nm is related to the ^3^[FAD˙^–^–MOPS˙^+^] ensemble. In the presence of oxygen, the rate of product formation slows down and a 20–25% decrease in the concentration of the anionic semiquinone species occurs, suggesting that oxygen competes with MOPS for ^3^FAD* (Fig. 5b). However, the decrease in the concentration of FAD˙^–^ is not as pronounced as one would expect from the oxygen reactivity with triplet-excited species, confirming the key role of MOPS in protecting active flavin species. This is further supported by the slower rate of recovery of the ground state upon turning off the excitation after 30 min (Fig. 5a). The distinctive properties of MOPS were further corroborated by experiments replacing MOPS with Tris-HCl buffer and/or EDTA (Fig. 5, Fig. S14b†). In Tris-HCl, the rate of formation of FAD˙^–^ is significantly lower compared to that measured in MOPS, and its steady-state concentration is 15–20% lower (Fig. S14b†). The use of EDTA as an ED increases the reaction rate but leads to an even larger drop in steady-state population, up to 40% less than that of MOPS (Fig. 5b).

**Fig. 6:**
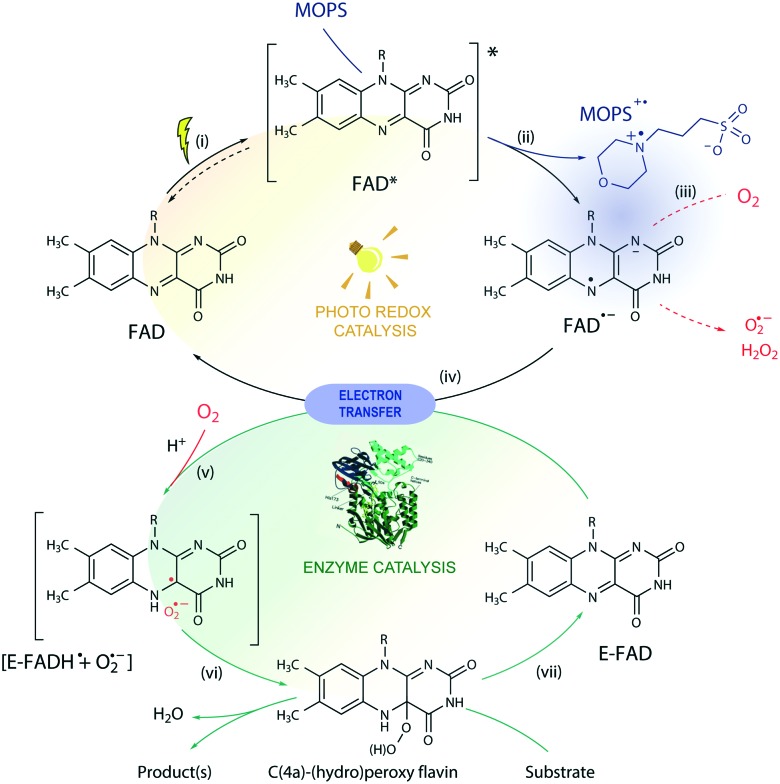
Proposed mechanism for the photobiocatalysis arbitrated by MOPS. In the photoredox cycle: (i) the oxidized FAD is excited by light to ^1,3^FAD*, capable of (ii) oxidizing MOPS. The resulting FAD semiquinone (FAD˙^–^) can (iii) generate ROS in the presence of O_2_*via* electron transfer or (iv) reduce the oxidized FAD bound to the enzyme (E-FAD), regenerating its oxidized form FAD. In the biocatalytic cycle, (v) the resulting caged radical pair [E-FADH˙ + O_2_˙^–^] originates (vi) C(4a)-(hydro)peroxyflavin, responsible for the conversion of the substrate, and the E-FAD is regenerated (vii). The ROS produced may induce enzyme deactivation, which is minimized in the presence of MOPS due to stabilization of the FAD semiquinone *via* formation of a ^1,3^[FAD˙^–^–MOPS˙^+^] ensemble (in purple). For simplicity, protonation equilibria are not shown.

Based on these results, we propose a general mechanism for stabilization of flavoproteins by MOPS (Fig. 6). Photoexcitation of the FAD cofactor produces ^1^FAD* that undergoes fast intersystem crossing producing ^3^FAD*. Excitation of FAD in the presence of MOPS results in the spin-correlated radical–ion pairs ^1^[FAD˙^(↑)–^–MOPS˙^(↓)+^] and ^3^[FAD˙^(↑)–^–MOPS˙^(↑)+^]. The long-lived ^3^[FAD˙^–^–MOPS˙^+^] precludes (or delays) the reduction of oxygen to ROS by FAD˙^–^ that could damage flavin oxidoreductases, such as CHMO_Acineto_.^28^

Enzyme activation depends on the transfer of reducing equivalents to the enzyme-bound FAD (E-FAD). Both E-FADH^–^ and E-FADH_2_ are expected to react with molecular oxygen forming the C(4a)-hydroperoxide adduct responsible for the Baeyer-Villiger oxidation, as proposed elsewhere.^29^ The reduction of E-FAD by 2e^–^ in the presence of H^+^ results in E-FADH^–^ which, in the presence of oxygen, produces the caged radical pair ^1,3^[E-FADH˙ + O_2_˙^–^].^10^ The singlet ensemble can undergo associative recombination in the presence of H^+^ producing E-FADH–OOH, which in the presence of a substrate leads to oxidation but can produce H_2_O_2_ and E-FAD in the uncoupled reaction.^10^,^30^ Photoreduction of FAD by amines such as EDTA and MOPS is expected to transfer 1e^–^, thus producing the ^1,3^[FAD˙^–^–amine˙^+^] or ^1,3^[FADH˙ + amine˙^+^] radical ion pair ensembles. Hence, ^1,3^[FAD˙^–^–MOPS˙^+^] must reduce E-FAD by 1e^–^, producing E-FADH^–^ necessary for the enzyme catalysis, regenerating the free FAD, and oxidizing MOPS. It is possible that the ^3^[FAD˙^–^–MOPS˙^+^] ensemble is very close to the flavin in the active site of the enzyme, as implied by crystallographic data of flavin oxidoreductases obtained in the presence of MES showing the internalized morpholine.^19^,^31^ Nevertheless, the electron transfer from the enzyme periphery cannot be ruled out considering the mechanism proposed based on magnetoreception by FAD tryptophan spin-correlated radical–ion pairs.^26^,^32^

## Conclusions

Flavins are versatile photosensitizers with promising application in the environmentally friendly and economically attractive photobiocatalysis field. Nevertheless, the formation of ROS under aerobic conditions restricts the expansion of this field to flavin-dependent enzymes that require oxygen for catalysis. MOPS goes far beyond a buffer and a sacrificial electron donor for photobiocatalysis. In fact, it stabilizes the triplet excited state and the radical anion semiquinone of FAD and consequently, prevents the formation of ROS under aerated conditions without affecting the enzymatic activity. The use of morpholine-based buffers in photobiocatalysis, especially MOPS, may also compromise the reliability of studies involving other sacrificial electron donors. The use of redox enzymes for solar chemical synthesis has been limited due to poor electron transfer kinetics between enzymes and the source of photoexcited electrons. However, prolonged life-time of the excited state of flavins and reduction of the formation of ROS by MOPS might be applicable in other systems involving light. Further details on the dynamic aspects of the formation and interchange of spin-correlated species may contribute to elucidation of the role of triplet excited states in the formation of ROS in processes involving flavin oxidoreductases, essential in biological processes implicated in diseases.^33^

## Conflicts of interest

There are no conflicts to declare.

## Supplementary Material

Supplementary information
