# Supplementary material for: Morpholine-based buffers activate aerobic photobiocatalysis via spin correlated ion pair formation
Source: Catal Sci Technol. 2019 Feb 11;9(6):1365–71. doi: 10.1039/c8cy02524j (PMC6468414; doi:10.1039/c8cy02524j)
Supplement: Supplementary file 1 [file CY-009-C8CY02524J-s001.pdf]

Electronic Supplementary Material (ESI) for Catalysis Science & Technology.
This journal is © The Royal Society of Chemistry 2019

Electronic Supplementary Information

Morpholine-based buffers activate aerobic photobiocatalysis via spin correlated ion pair formation

Leticia C. P. Gonçalves[1,*], Hamid R. Mansouri[1], Erick L. Bastos[2], Mohamed Abdellah[3,4], Bruna S. Fadiga[2,3], Jacinto Sá[3,5], Florian Rudroff[1], and Marko D. Mihovilovic[1]

[1]Institute of Applied Synthetic Chemistry, TU Wien, Vienna, Austria.

[2]Department of Fundamental Chemistry, Institute of Chemistry, University of São Paulo, São Paulo, Brazil.

[3]Physical Chemistry Division, Department of Chemistry, Ångström Laboratory, Uppsala University, 75120 Uppsala, Sweden.

[4]Department of Chemistry, Qena Faculty of Science, South Valley University, 83523 Qena, Egypt.

[5]Institute of Physical Chemistry, Polish Academy of Sciences, 01-224 Warsaw, Poland.

[*]Corresponding author: Getreidemarkt 9/163, 1060 Vienna, Austria; e-mail: leticia.goncalves@tuwien.ac.at

CONTENTS

Experimental details 3
General considerations 3
Growth of Bacterial Cells for Enzyme Expression and Isolation 3
Enzyme Purification 3
Enzyme Activity and Stability Measurements 4
Light Source 5
Gas Chromatography (GC) Analysis 5
Determination of Hydrogen Peroxide (H_2O_2) 5
Synthesis of 2,2,6-trimethyl-1,4-cyclohexanedione (levodione) 6

Preparation and isolation of 2-phenyl-ε-caprolactone 6

SUPPLEMENTARY FIGURES 8

GC chromatograms of the reduction of ketoisophorone (1a) by XenB 8

GC chromatograms of the BV oxidation of cyclohexanone (2a) by $CHMO_{Acineto}$ 9

Effect of the concentration of $CHMO_{Acineto}$, FAD and $NADP^+$ on the photo-induced enzyme catalyzed BV oxidation of ε-caprolactone (2a) 10

Photoinduced Baeyer-Villiger oxidation of cyclohexanone (2a) by $CHMO_{Acineto}$ 11

Photoinduced BV oxidation of 2-phenylcyclohexanone (3a) by $CHMO_{Acineto}$ 12

Photoinduced BV oxidation of bicyclo[3.2.0]hept-2-en-6-one (4a) by $CHMO_{Acineto}$ 13

Control experiments in the absence of $CHMO_{Acineto}$ upon irradiation 14

Effect of the buffer on the stability of $CHMO_{Acineto}$ 15

Formation of H_2O_2 upon daylight irradiation of FAD 16

Hydrogen peroxide calibration curve (ABTS/HRP system) 17

Effect of the electron donor on the absorption spectra of FAD upon daylight irradiation 18

Transient absorption measurements in aerated solution 19

Steady-state absorption controls of FAD and MOPS under irradiation 20

Steady-state absorption measurements of FAD under irradiation 21

SUPPLEMENTARY TABLES 22

SUPPLEMENTARY REFERENCES 23

Experimental details

General considerations

All chemicals were obtained from Sigma Aldrich (highest purity available) and used without further purification unless otherwise stated.

Buffer solutions

Photochemical experiments were carried out in one of the following solutions, which were prepared in dH_2O and the pH set to 7.5 with 1 M NaOH or 1 M HCl:

A) 100 mM MOPS buffer (3-(N-Morpholino)propanesulfonic acid, > 99.5%);

B) 100 mM Tris-HCl buffer (Tris base, > 99.0%);

C) 100 mM sodium phosphate buffer (Na_2HPO_4 / NaH_2PO_4, > 99.0%);

D) 100 mM HEPES buffer (4-(2-hydroxyethyl)piperazine-1-ethanesulfonic acid, > 99.5%);

E) 100 mM MES buffer (2-(N-Morpholino)ethanesulfonic acid), > 99.0%);

F) 100 mM morpholine (tetrahydro-1,4-oxazine, > 99.5%).

Growth of Bacterial Cells for Enzyme Expression and Isolation

$CHMO_{Acineto}$ (cyclohexanone monooxygenase from *Acinetobacter calcoaceticus* NCIMB 9871) and XenB (enoate reductase from *Pseudomonas sp.*) were expressed in *E. coli* strain BL21(DE3) according to previously published procedures.[1, 2] Lysogeny broth (LB) medium (6 mL) supplemented with ampicillin (100 µg mL^{-1}) was inoculated with either *E.coli* BL21(DE3) pET22b(+)_$CHMO_{Acineto}$[3] or pGASTON_XenB[4]. These were grown over-night at 37 ºC in an orbital shaker operated at 200 rpm. The cultures were transferred to 1 L baffled Erlenmeyer flask containing 250 mL LB/ampicillin and shaken at 200 rpm and 37 ºC for approximately 2.5 h to a final optical density at 590 nm of approx. 0.6. Isopropyl β-D-thiogalactopyranoside (IPTG) was added to a final concentration of 50 µM ($CHMO_{Acineto}$) and the flask was incubated for 18–22 h at 20 ºC. L-rhamnose, 0.2%, was added for the induction of XenB, which was incubated for 18–22 h at 25 ºC. Cells were harvested by centrifugation (4000 × g, 4 ºC, 15 min).

Enzyme Purification

Cell pellets were re-suspended in 50 mM Tris-HCl buffer, pH 7.5, containing 0.1 mM phenylmethylsulfonyl fluoride (PMSF) and 0.1 mM FAD ($CHMO_{Acineto}$) or 0.1 mM FMN (XenB). Cells were placed on ice and sonicated using a Bandelin KE76 sonotrode connected to a Bandelin

Sonoplus HD 3200 in 9 cycles (5 s pulse, 55 s break, amplitude 50%). Cell debris were removed by centrifugation (15000 × g, 4 ºC, 45 min) and the clarified supernatants containing the polyhistidine-tagged $CHMO_{Acineto}$ or XenB wild-type enzymes were loaded on a Ni^{2+}-Sepharose HP affinity column (5 mL, GE Healthcare bioscience) equilibrated with 50 mM Tris-HCl buffer, pH 7.5, containing 0.5 M NaCl and 0.1 mM FAD ($CHMO_{Acineto}$) or FMN (XenB). Enzymes were eluted in 4 column volumes within a linear gradient from 25 to 250 mM imidazole in 50 mM Tris-HCl buffer, pH 7.5, containing 0.5 M NaCl and 0.1 mM FAD ($CHMO_{Acineto}$) or FMN (XenB). Fractions containing the enzymes were identified by SDS-PAGE analysis, pooled, desalted, washed with 50 mM Tris-HCl (pH 7.5) containing 0.1 mM FAD ($CHMO_{Acineto}$) or FMN (XenB) and concentrated by ultrafiltration by using ultra centrifugal tubes with a cut-off of 10 kDa. Protein concentrations were determined by the dye-binding method of Bradford using a pre-fabricated assay (Bio-Rad) and bovine serum albumin as the calibration standard. Buffer exchange was done using Amicon Ultra Centrifugal Filters (10 kDa, 0.5 mL, Millipore) for experiments performed in buffers other than 50 mM Tris HCl (pH 7.5).

Enzyme Activity and Stability Measurements

Enzyme activities were measured by monitoring the substrate-dependent decrease in NADPH absorbance at 340 nm ($\varepsilon^{340} = 6.22\ mM^{-1}\ cm^{-1}$) in 100 mM Tris-HCl or 100 mM MOPS (pH 7.5). Standard assays for the activity measurement of $CHMO_{Acineto}$ contained 0.05 µM enzyme, 100 µM NADPH and 0.5 mM cyclohexanone. Measurements were done according to a previously published procedure[1]. Oxidation of NADPH was followed for 120 s at 30 ºC in a Shimadzu spectrophotometer (UV-1800) featuring a thermo-controlled 6-cell positioner (CPS-240A). All kinetic measurements were performed in triplicate unless otherwise stated. Enzyme activity is defined as the amount of enzyme that oxidizes 1 µmol NADPH per minute under the specified conditions. Specific activities were calculated from the observed rate constants (k_{obs}), which were obtained by fitting the initial rate of the absorbance changes to a linear regression (Origin 2017 for Windows). Stability measurements were performed by incubating 10 µM enzyme at 30 ºC in 100 mM Tris-HCl or 100 mM MOPS, pH 7.5, containing 100 µM FAD ($CHMO_{Acineto}$). Aliquots were taken at different time points and added to a cuvette containing 0.1 mM NADPH and 0.5 mM substrate to test for catalytic activity. Experimental data were fitted to an exponential decay

equation using the Origin Pro software (Origin 8.5 for Windows). Data are reported as $\bar{x} \pm 1$ SD (n=3) unless otherwise stated.

Light Source

For the daylight lamp experiments, the lamp was placed at 20 cm distance from the 96 well-plate. The light intensity on the plate level was measured with an Ocean Optics Spectrophotometer (USB2000+) at 450 nm for the daylight lamp (28 μW/cm^2). The region where the light intensity remained the same was determined and the experiments performed in the area where the amount of energy was constant.

Gas Chromatography (GC) Analysis

For GC analysis, 50 μL of the reaction mixture were added to 1.5 mL Eppendorf tubes containing 150 μL ethyl acetate supplemented with 1 mM methyl benzoate as internal standard. Samples were vortexed at maximum speed (IKA Vortex 4 basic) for 30 s and centrifuged for 1 min (VWR Silverstar bench top centrifuge). The organic layer was transferred into a new 1.5 mL Eppendorf tube and dried over Na_2SO_4. After centrifugation, the supernatant was transferred to a 1.5 mL GC glass vial equipped with a 0.1 mL micro-insert and subjected to GC analysis.
GC achiral analyses were performed with a BGB5 (30 m x 0.25 mm ID, 0.25 μm film) on a Thermo Finnigan Focus GC / DSQ II (Thermo Scientific) and GC chiral analysis, with a BGB175 column (30 m x 0.25 mm ID, 0.25 μm film) on a ThermoQuest Trace GC 2000 (Thermo Scientific). The amount of product was quantified from the peak areas using an internal standard (1 mM methyl benzoate) and corrected using the response factor obtained from the linear regression adjust of the calibration curve, unless otherwise stated.

Determination of Hydrogen Peroxide (H_2O_2)

Hydrogen peroxide (H_2O_2) was measured by the ABTS based assay. This assay quantifies the production of H_2O_2 in the presence of horseradish peroxidase (HRP), which uses the H_2O_2 to convert the colorless ABTS reactant to its oxidized turquoise form. The amount of H_2O_2 in the samples irradiated with the daylight lamp (300 W, Osram) containing FAD (100 μM) in 100 mM MOPS, in 100 mM Tris-HCl or in water, pH 7.5, as well as control samples without any flavin. For that, 50 μL aliquots were taken over time and added to 2 mM ABTS and 5.8 U/mL HRP to a final volume of 500 μL. The absorption spectrum was recorded for each sample and the amount

of H_2O_2 quantified after calibration curve with commercial H_2O_2 at 734 nm (intercept = 0.067; slope = 0.02702 ± 0.0008, Adj-R^2 > 0.99).

Synthesis of 2,2,6-trimethyl-1,4-cyclohexanedione (levodione)

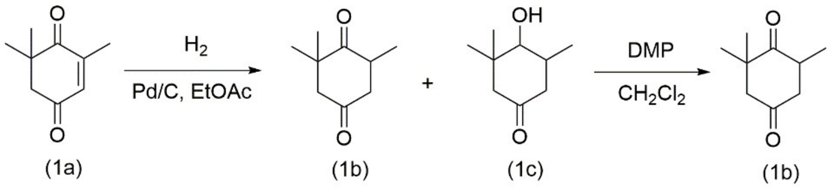

Ketoisophorone **(1a)** (500 mg, 3.3 mmol, 1 eq.) was placed in a 3-neck-round bottomed flask and ethyl acetate (5 mL) was added. 75 mg of Palladium on charcoal (75 mg, 10%$_{w/w}$ were added to the mixture and the mixture was evacuated and purged with argon three times. After the last purging step, the evacuated state was maintained and the Schlenk-line tube was exchanged with a hydrogen balloon. The apparatus was flushed with H_2 and the mixture was stirred overnight. Upon reaction completion (monitored by GC/MS analysis), the reaction mixture was filtered over Celite and the solvent was removed under reduced pressure (428 mg crude yield). The crude product (100 mg) was mixed with Dess-Martin-Periodinane (DMP, 300 mg, 0.7 mmol, 1.06 eq. *vs* **1c**) in CH_2Cl_2 and stirred for 15 minutes at room temperature. After completion (monitored by TLC), the reaction was extracted with a 10%$_{w/v}$ $Na_2S_2O_3$ solution followed by extraction with a saturated solution of NH_4Cl. The aqueous layers were re-extracted with CH_2Cl_2 and the resulting organic layer was dried over Na_2SO_4 and filtered through silica. The solvent was removed under reduced pressure resulting in a beige solid (**1b**, 42 mg).

^{1}H-NMR (400 MHz, $CDCl_3$): δ(ppm)= 1.14 (s, 3H), 1.17 (d, 3H, J^3= 6.5 Hz), 1.21 (s, 3H), 2.30-2.42 (m, 1H), 2.54 (d, 1H, J^2= 15.5 Hz), 2.72- 2.83 (m, 1H), 2.96- 3.08 (m, 1H). Spectrum in accordance to the literature.[5]

^{13}C-NMR (100 MHz, $CDCl_3$): δ(ppm)= 14.6, 25.6, 26.5, 39.8, 44.2, 44.9, 52.8, 208.0, 214.1. Spectrum in accordance to the literature.[6]

Preparation and isolation of 2-phenyl-ε-caprolactone

The reference compound 2-phenyl-ε-caprolactone (0.6 mmol) was obtained by the biotransformation of 2-phenyl-cyclohexanone (1.2 mmol) with CPDMO (cyclopentadecanone monooxygenase from *Pseudomonas sp.)*.[7] LB medium (6 mL) supplemented with ampicillin (100 µg mL^{-1}) was inoculated with *E.coli* TOP10 pBAD_CPDMO and cells were grown over-

night at ºC in an orbital shaker operated at 200 rpm. The cultures were transferred to 1 L baffled Erlenmeyer flask containing 250 mL TB/ampicillin and shaken at 200 rpm and 37 ºC for 2.5 h to a final optical density at 590 nm of approx. 0.6. Cells were induced with $10\%_{w/v}$ arabinose. The substrate 2-phenylcyclohexanone (0.62 mmol, in dioxane) was added 20 min after induction in the presence of 1 mM cyclodextrin. After 3 h, cells were centrifuged (6000 rpm, 15 min) and the product extracted from the supernatant with petrol ether (5 × 100 mL) and the organic layer washed with brine (1 × 150 mL). The crude yellow oil was purified by medium pressure liquid chromatography using a petrol ether: ethyl acetate mixture (50% yield). The spectral data are in agreement with the literature values.[8]

SUPPLEMENTARY FIGURES

GC chromatograms of the reduction of ketoisophorone (1a) by XenB

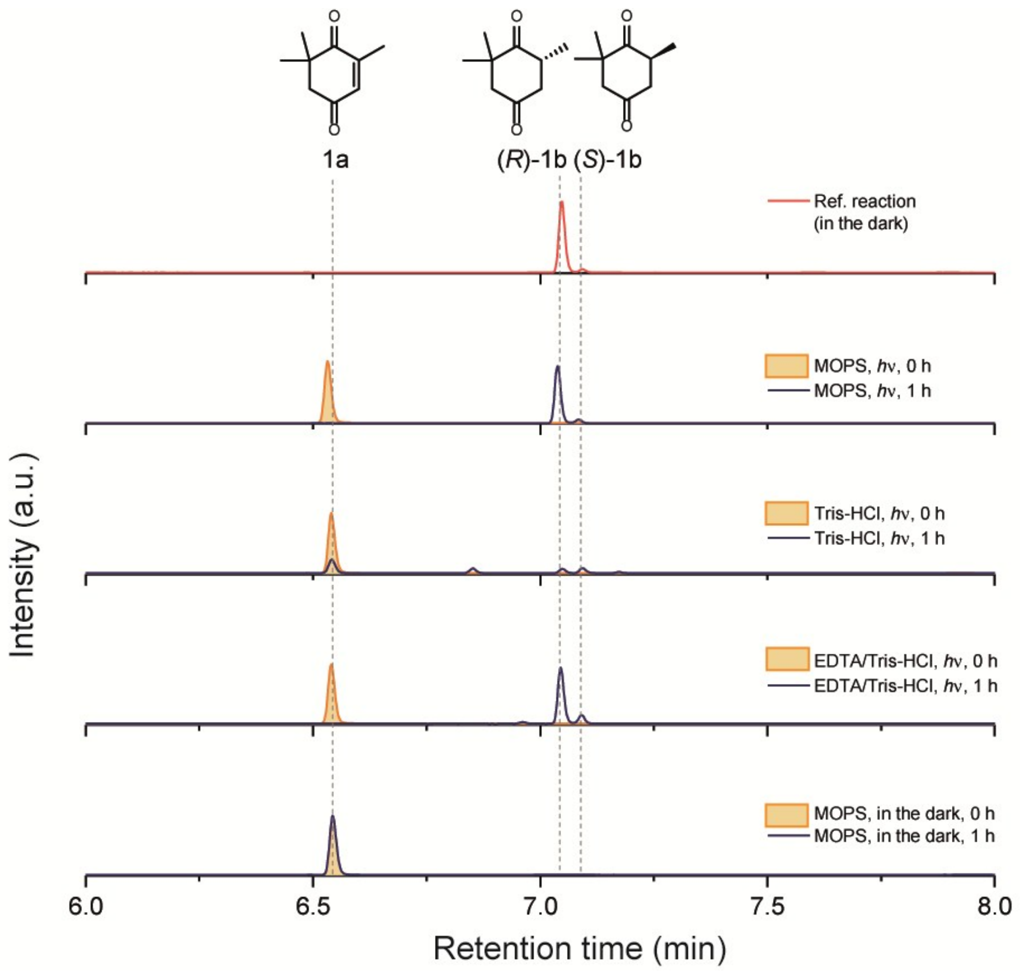

Figure S1. Gas chromatography (GC) chromatograms of (from top to bottom): reference reaction employing the enzymatic regeneration system GDH/Glucose/NADP$^+$ after 1 h in 100 mM MOPS, pH 7.5; standard reaction in 100 mM MOPS, pH 7.5; standard reaction in 100 mM Tris-HCl, pH 7.5; standard reaction in 25 mM EDTA/ 100 mM Tris-HCl, pH 7.5; standard reaction in the dark in 100 mM MOPS, pH 7.5. Standard reaction: 10 µM XenB, 100 µM FMN, 1 mM ketoisophorone (**1a**). Irradiation source: daylight lamp (300 W, Osram). Time points: 0 and 1 h.

GC chromatograms of the BV oxidation of cyclohexanone (2a) by CHMO*Acineto*

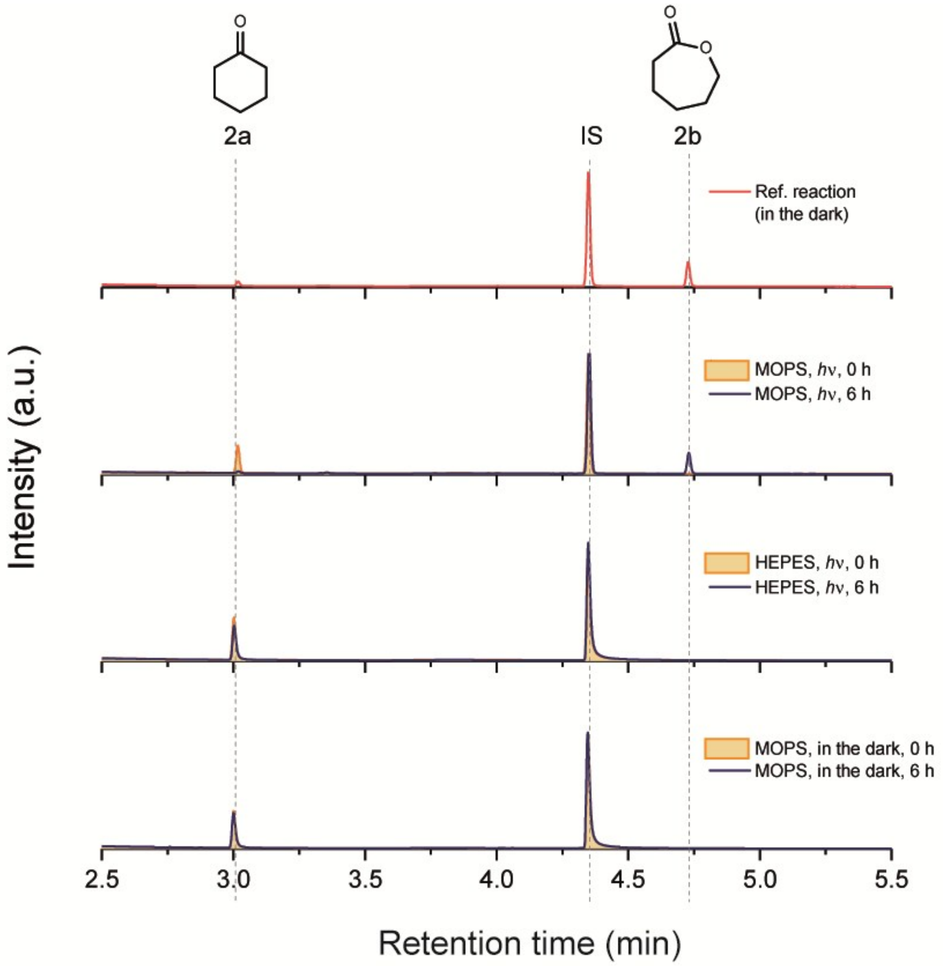

Figure S2. Gas chromatography (GC) chromatograms of (from top to bottom): reference reaction employing the enzymatic regeneration system GDH/Glucose/$NADP^+$ after 6 h in 100 mM MOPS, pH 7.5; standard reaction in 100 mM MOPS, pH 7.5, 0 and 6 h of irradiation; standard reaction in 100 mM HEPES, pH 7.5, 0 and 6 h of irradiation; standard reaction in 100 mM MOPS, pH 7.5, 0 and 6 h in the dark. Standard reaction: 10 µM $CHMO_{Acineto}$, 100 µM FAD, 250 µM $NADP^+$, 1 mM cyclohexanone (**2a**). Irradiation source: daylight lamp (300 W, Osram). IS: internal standard, 1 mM methyl benzoate.

The possible H_2O_2-shunt for the BV oxidation has been previously excluded.[9] Yet, product **2b** was not detected after irradiation of the system **2a**/$CHMO_{Acineto}$ in HEPES buffer, known to generate H_2O_2[10].

Effect of the concentration of CHMO*Acineto*, FAD and NADP+ on the photo-induced enzyme catalyzed BV oxidation of ε-caprolactone (2a)

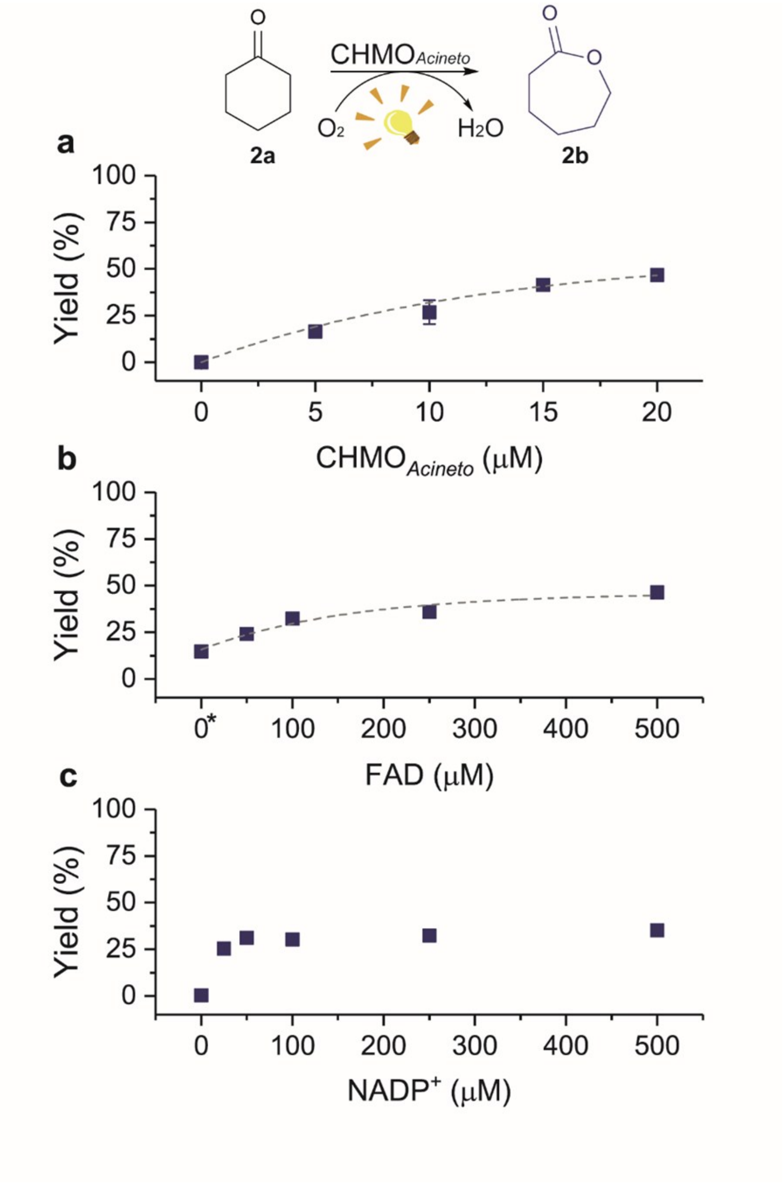

Figure S3. Photoinduced enzyme catalyzed Baeyer-Villiger oxidation of cyclohexanone (1 mM). (a) Effect of the enzyme concentration, CHMO*Acineto* (0 – 20 μM) in the presence of 100 μM FAD and 250 μM NADP+, (b) effect of the concentration of FAD (0* – 500 μM) employing 10 μM CHMO*Acineto* in the presence of 250 μM NADP+ and (c) effect of the concentration of NADP+ (0 – 500 μM) employing 10 μM CHMO*Acineto* in the presence of 100 μM FAD. Reactions in were performed in 100 mM MOPS (pH 7.5). Irradiation time: 1 h with the daylight lamp (300 W, Osram). CHMO*Acineto* was supplemented with FAD for purification and storage to improve stability, therefore, it is not possible to determine the yield in FAD free condition and FAD = 0* represents no addition of extra FAD.

Note: overall reaction performance strongly depends on the experimental set up. By increasing the sampling frequency – thereby opening the reaction vessel and momentarily ceasing illumination – lead to reduced yield and reaction velocity. After 1 h irradiation, the yield was reduced to 40% in comparison to the reaction that was not sampled (**Fig. 2b)**.

Photoinduced Baeyer-Villiger oxidation of cyclohexanone (2a) by CHMO$_{Acineto}$

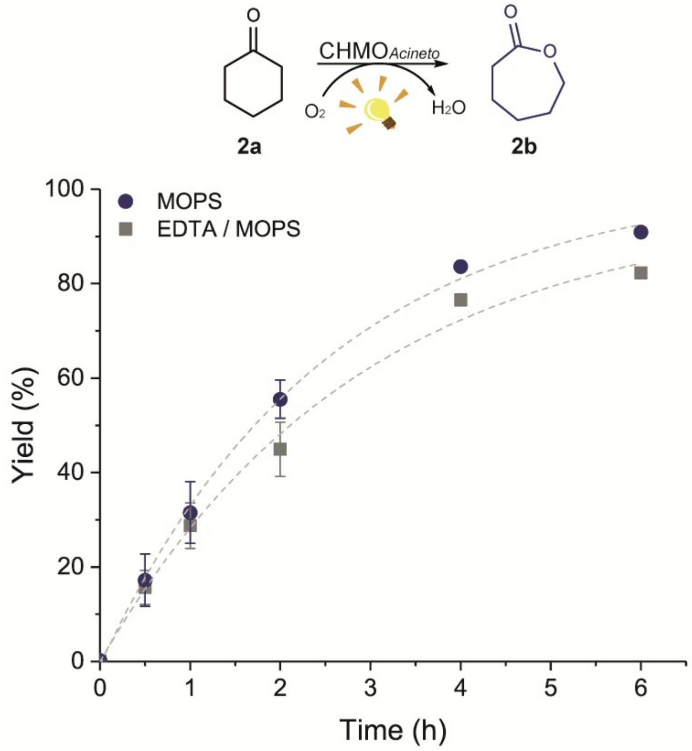

Figure S4. Photoinduced enzyme catalyzed Baeyer-Villiger oxidation of cyclohexanone (1 mM) employing CHMO$_{Acineto}$ (10 µM) as biocatalyst and FAD (100 µM) as mediator. ε-Caprolactone (**2b**) yield over time of reactions irradiated with the daylight lamp (300 W, Osram) for 0.5, 1, 2, 4, and 6 h in 100 mM MOPS (pH 7.5) in the presence and in the absence of EDTA (25 mM), containing 250 µM $NADP^+$. Product formation rate: 0.31 ± 0.02 mM h^{-1}; initial turnover frequency (TOF): 32 h^{-1} (initial 60 min). Note that the addition of EDTA to the system had no effect on reaction rate and yield, evidencing no synergism between EDTA and MOPS.

Photoinduced BV oxidation of 2-phenylcyclohexanone (3a) by CHMO$_{Acineto}$

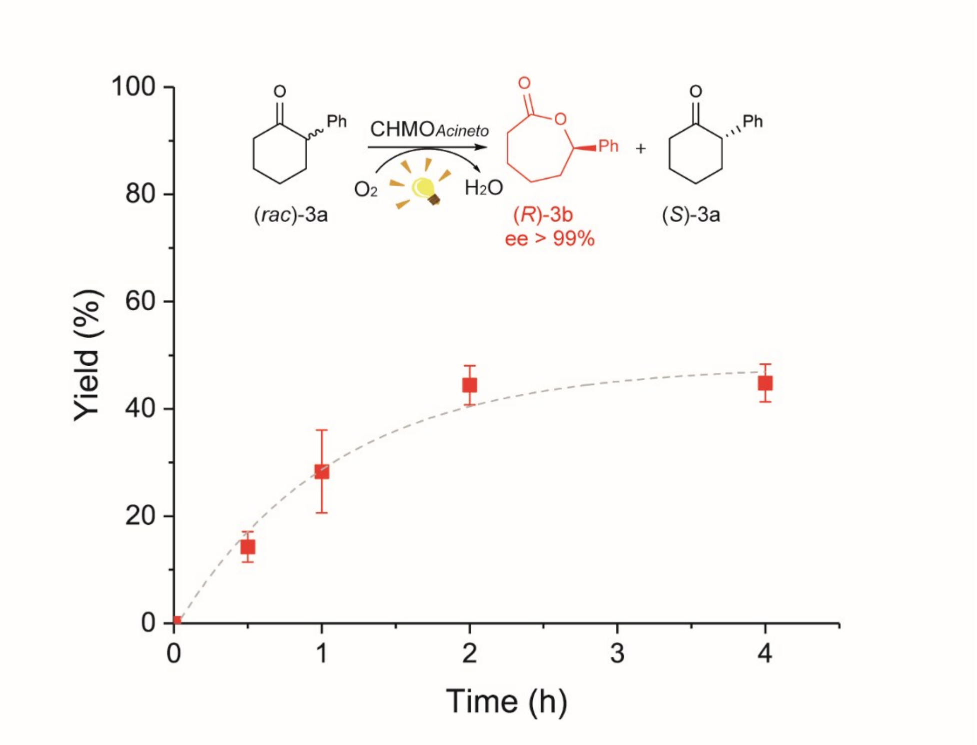

Figure S5. Photoinduced enzymatic Baeyer-Villiger oxidation of 2-phenyl-cyclohexanone (1 mM) employing CHMO$_{Acineto}$ (10 µM) as biocatalyst and FAD (100 µM) as mediator. Reaction mixtures containing 250 µM $NADP^+$ were irradiated with the daylight lamp (300 W, Osram) for 0.5, 1, 2, and 4 h in 100 mM MOPS (pH 7.5). TOF = 28.3 h^{-1}. The reference reaction was performed using the enzymatic recycling system GDH/Glu. Yield (48%) and enantiomeric excess (*ee* > 99%) are in agreement with the literature.[11] Note: product yield can reach maximum 50%.[11]

Photoinduced BV oxidation of bicyclo[3.2.0]hept-2-en-6-one (4a) by $CHMO_{Acineto}$

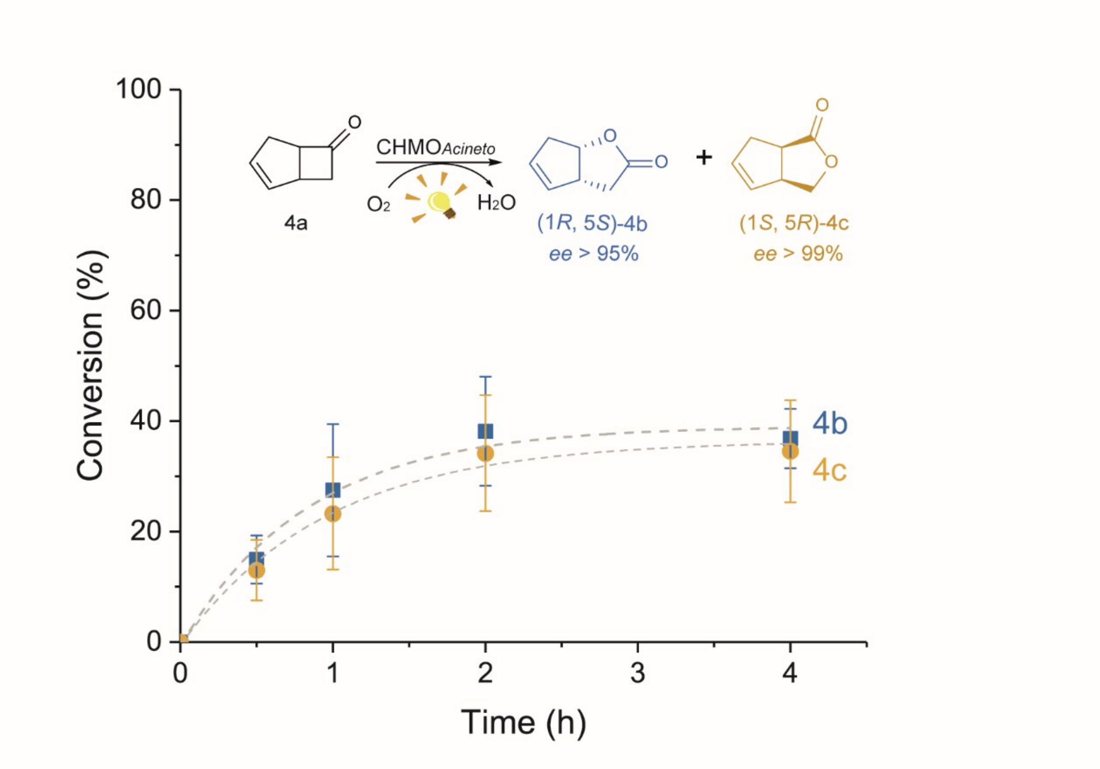

Figure S6. Photoinduced enzymatic Baeyer-Villiger oxidation of bicyclo[3.2.0]hept-2-en-6-one (1 mM) employing $CHMO_{Acineto}$ (10 µM) as biocatalyst and FAD (100 µM) as mediator. Reaction mixtures containing 250 µM $NADP^+$ were irradiated with the daylight lamp (300 W, Osram) for 0.5, 1, 2, and 4 h in 100 mM MOPS (pH 7.5) in 100 mM MOPS buffer (pH 7.5). TOF = 49.1 h^{-1}. The reference reaction was performed using the enzymatic recycling system GDH/Glu. Yield (**4b**: 51% and **4c**: 49%) and enantiomeric excess (**4b**: > 95% and **4c**: > 99%) of the reference reaction are in agreement with the literature.[12]

Control experiments in the absence of CHMO$_{Acineto}$ upon irradiation

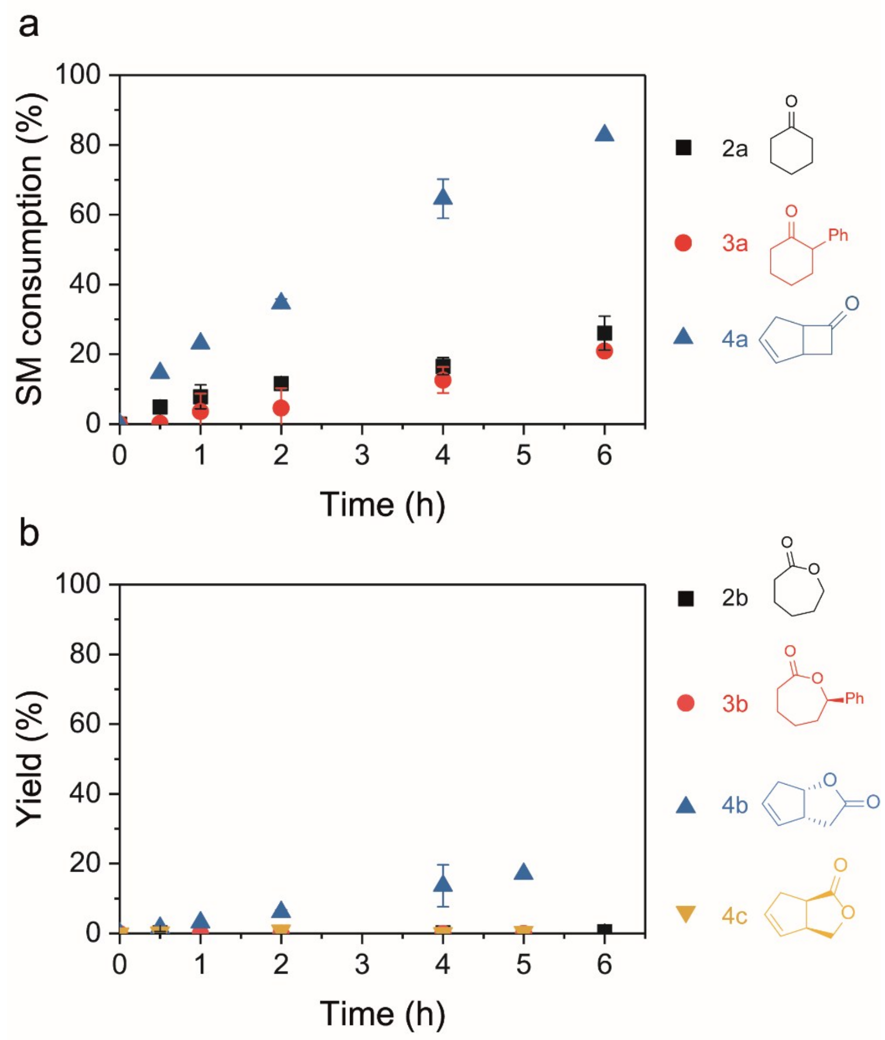

Figure S7. Photoreactions in MOPS buffer (100 mM, pH 7.5) in the absence of biocatalysts. (a) Starting material (SM) consumption after different irradiation time points of cyclohexanone (**2a**, □), 2-phenyl-cyclohexanone (**3a**, ○) or bicyclo[3.2.0]hept-2-en-one (**4a**, Δ) in the presence of 100 µM FAD and 250 µM $NADP^+$ with the daylight lamp (300 W, Osram). (b) Product formation (GC-Yield in %) after different irradiation time points of the substrates shown in (a) at the same conditions. **4b** and **4c** are shown in terms of conversion.

Effect of the buffer on the stability of CHMO$_{Acineto}$

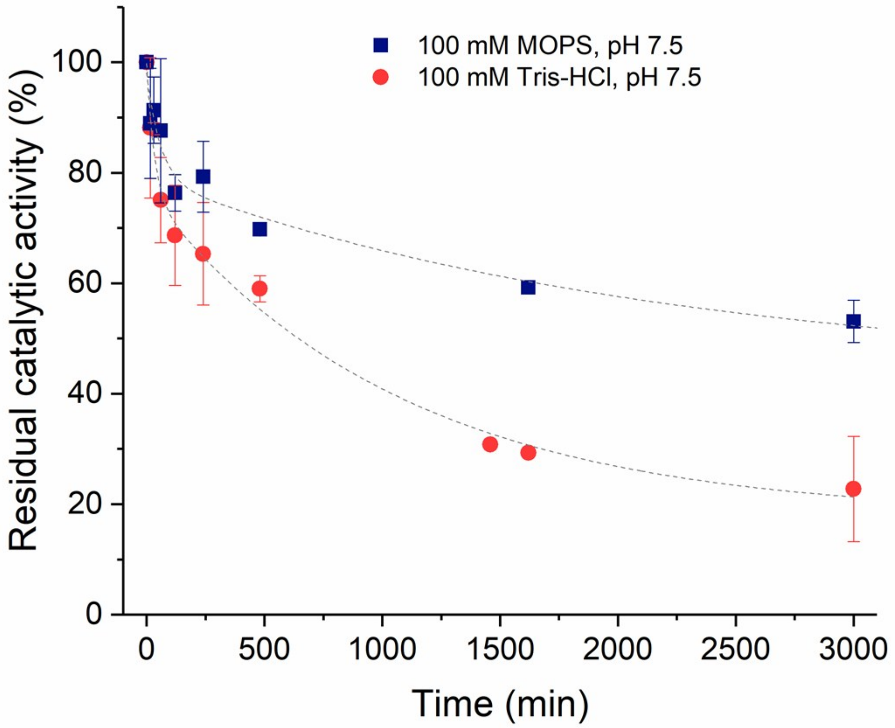

Figure S8. Effect of the buffer (MOPS *vs.* Tris-HCl) on the stability of CHMO$_{Acineto}$ (10 µM) at 30 ºC in the dark. CHMO$_{Acineto}$ was supplemented with FAD (100 µM) before incubation in 100 mM MOPS buffer, pH 7.5 (■) or in 100 mM Tris-HCl buffer, pH 7.5 (●). Enzyme activity was measured over time by the decrease in the NADPH absorption at 340 nm.

Formation of H_2O_2 upon daylight irradiation of FAD

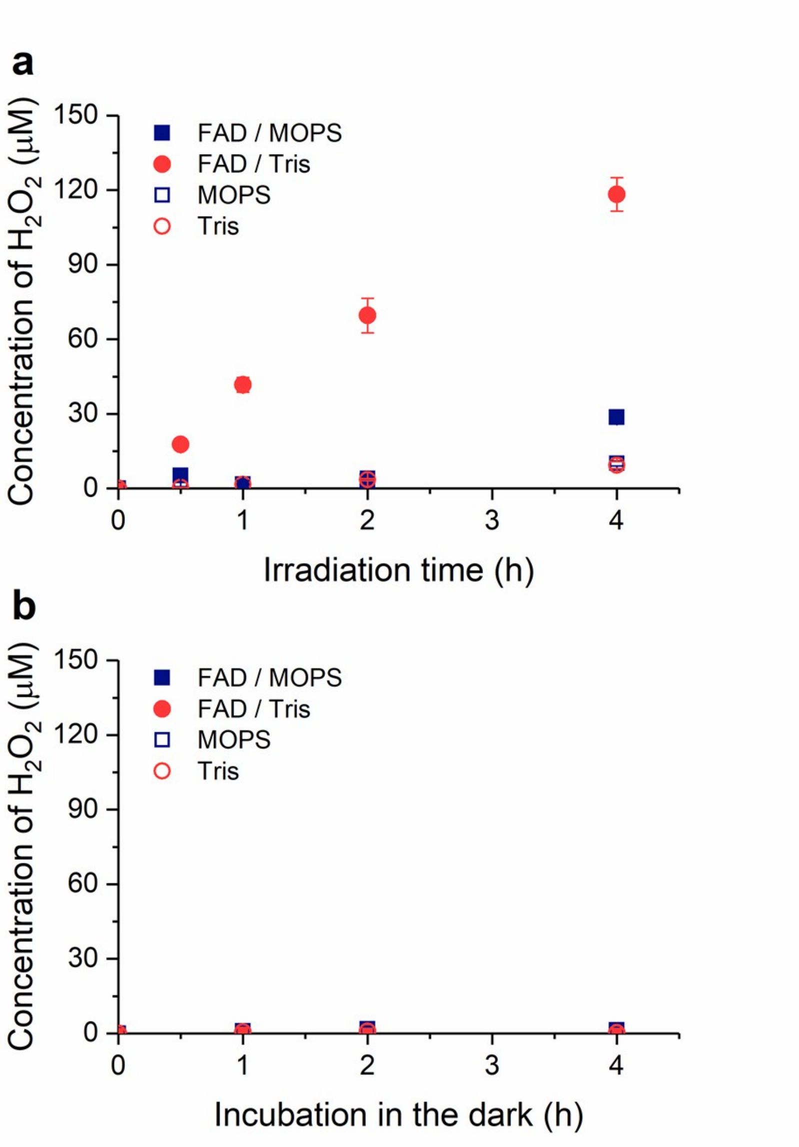

Figure S9. Concentration of Hydrogen peroxide (H_2O_2) generated over the time of (a) irradiation or (b) incubation in the dark of FAD (100 μM) in 100 mM MOPS, pH 7.5, and in 100 mM Tris-HCl, pH 7.5. Controls experiments in the absence of FAD are also shown in (a) and (b). Light source: Daylight lamp (300 W, Osram). The concentration was determined by the ABTS/HRP system (λ = 734 nm) using the calibration curve shown in the **Fig S10**. Samples with larger response than acceptable by the calibration curve were diluted for the measurement.

Hydrogen peroxide calibration curve (ABTS/HRP system)

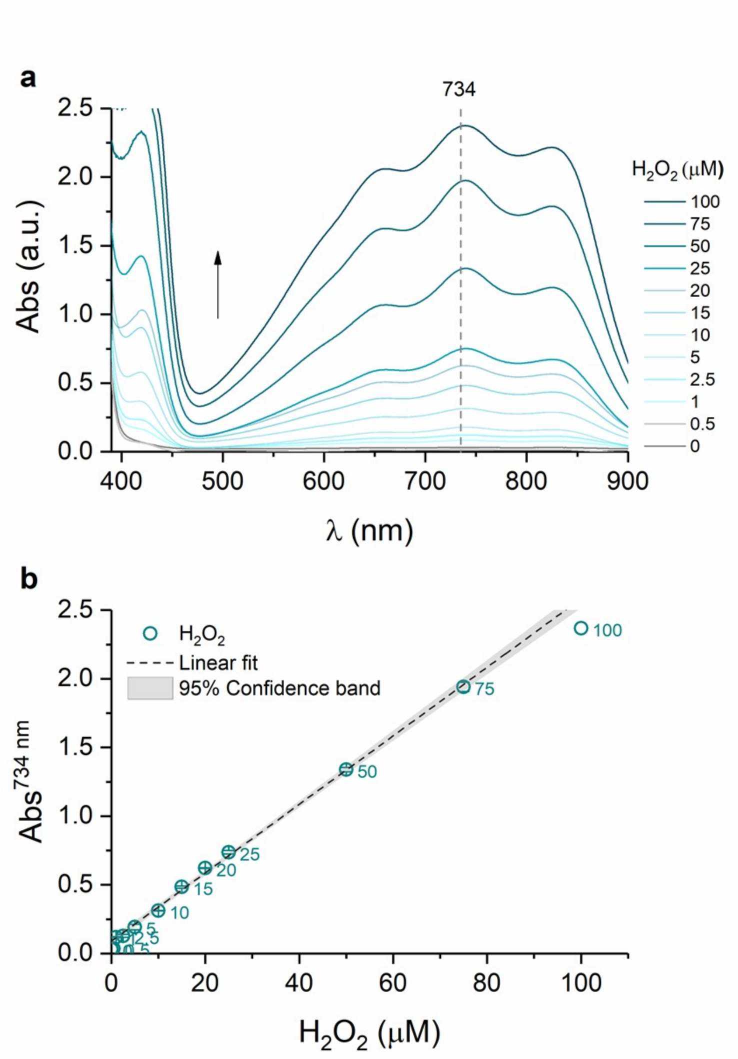

Figure S10. Calibration curve for the quantification of H_2O_2 using the ABTS/HRP colorimetric assay. (a) Absorption spectra of the $ABTS^{\bullet+}$ in the presence of H_2O_2 (0 – 100 μM). (b) Linear regression adjust of the absorption at 734 nm *vs* concentration of H_2O_2, slope = 0.0271 ± 0.0008 (intercept = 0.067), Adj-$R^2 > 0.99$. Note: data between 50 – 100 μM show absorbance intensity above the Beer-Lambert law but only 100 μM is out of the linear range, therefore excluded from the adjust. Samples were dilute to confirm the linearity of the regression when Abs > 1.0.

Effect of the electron donor on the absorption spectra of FAD upon daylight irradiation

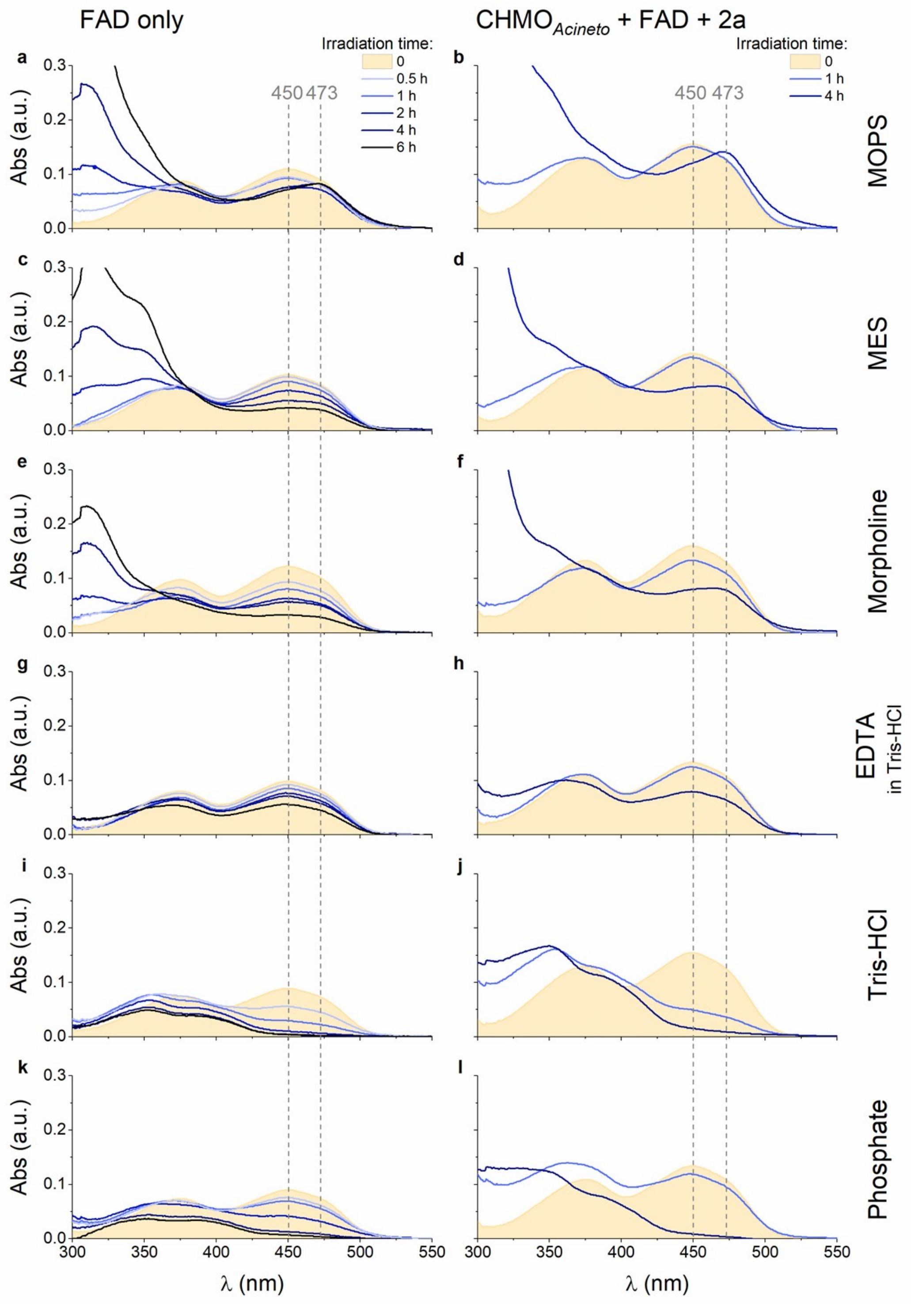

Figure S11. Effect of the electron donor (ED) on the absorption spectra of 100 µM FAD only (graphs on the left) and of 100 µM FAD during the photoinduced enzymatic Baeyer-Villiger oxidation of cyclohexanone (**2a**, 1 mM) to ε-caprolactone (**2b**) with CHMO$_{Acineto}$ (10 µM) containing 250 µM NADP$^+$ (graphs on the right). The samples were irradiated over time with the Daylight lamp (300 W, Osram) in the following solutions (100 mM, pH 7.5): MOPS buffer, MES buffer, morpholine, EDTA (25 mM) in Tris-HCl buffer, Tris-HCl buffer, and phosphate buffer.

Transient absorption measurements in aerated solution

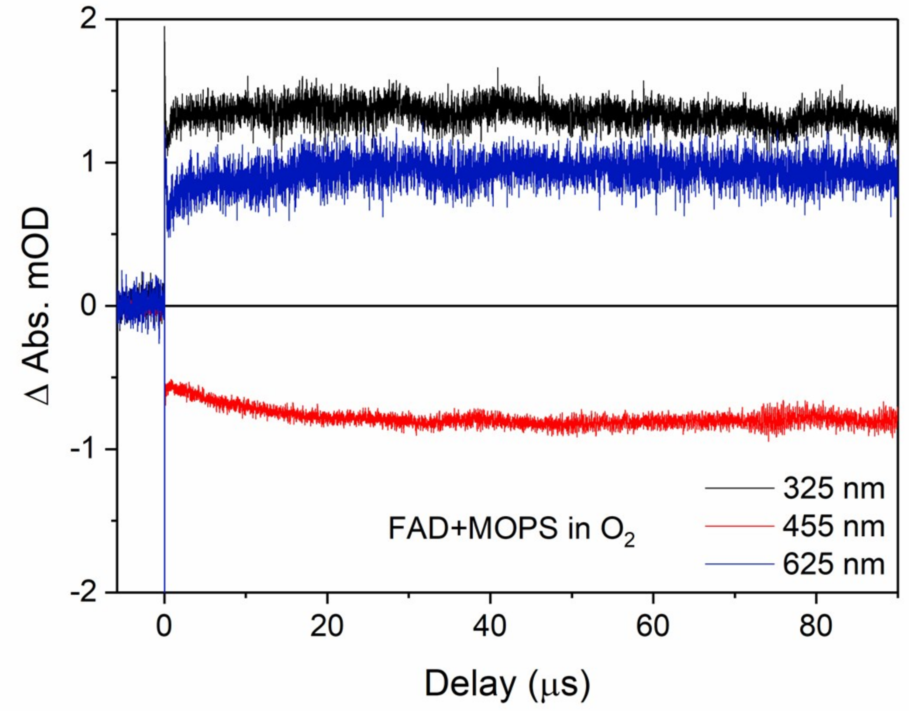

Fig S12. Kinetic trace extracted at 455 nm (FAD ground state absorption), 655 nm (FAD triplet state absorption) and 325 nm (FAD anionic semiquinone).

Steady-state absorption controls of FAD and MOPS under irradiation

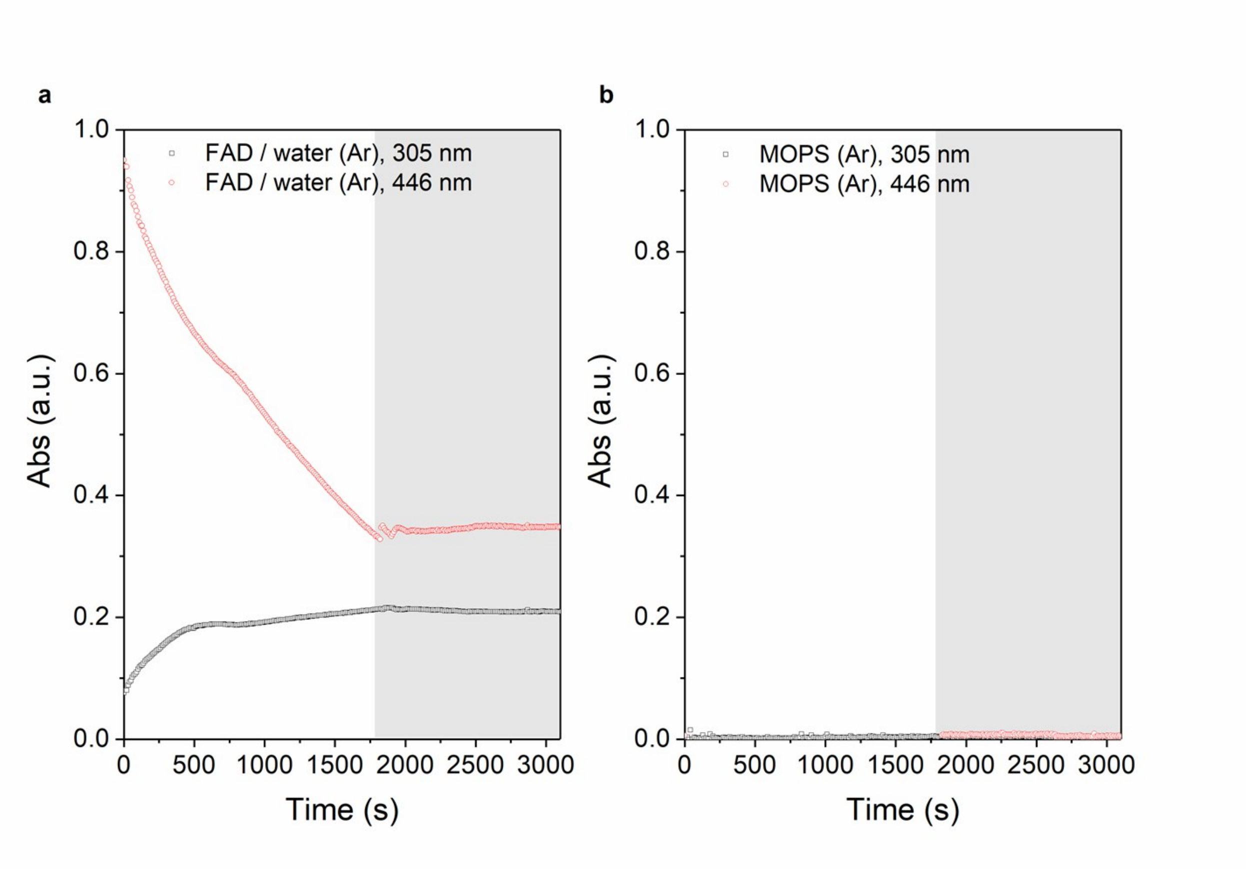

Fig. S13. Kinetic traces extracted from the absorption spectra of (a) FAD (100 µM) in water and (b) MOPS buffer pH 7.5 (100 mM) under light excitation (λ_{EX}= 445 nm). The kinetic trace at 455 nm is associated with FAD ground-state absorption, while the kinetic trace at 305 nm is ascribed to FAD anionic semiquinone.

Steady-state absorption measurements of FAD under irradiation

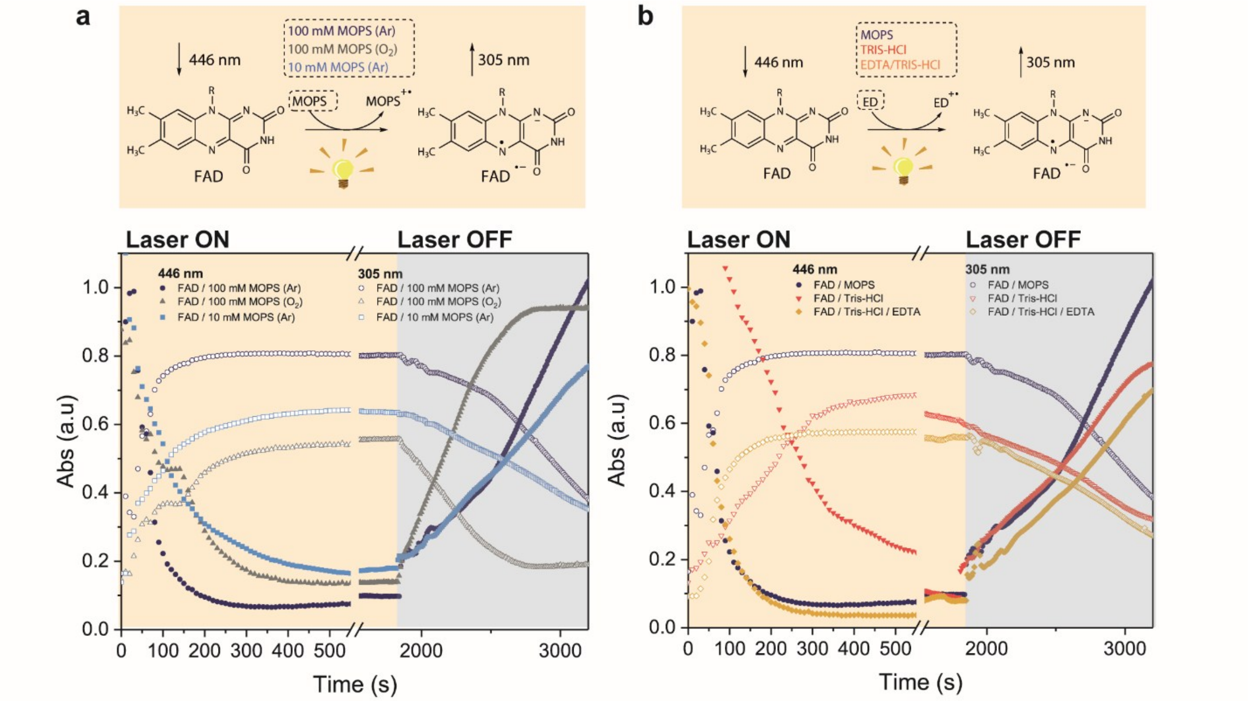

Fig. S14. Kinetic traces extracted from the absorption spectra of the reactions between FAD (100 μM) and buffers under light excitation (λ_{EX}= 445 nm). The kinetic trace at 455 nm is associated with FAD ground-state absorption, while the kinetic trace at 305 nm is ascribed to FAD anionic semiquinone. (a) effect of MOPS concentration (100 mM vs 10 mM) and molecular oxygen; and (b) buffer effect (Tris-HCl: 100 mM and EDTA: 25 mM). The pH of all solutions was set to 7.5.

SUPPLEMENTARY TABLES

Table S1. Enzyme activity (U/mg) in 100 mM MOPS and 100 mM Tris-HCl (pH 7.5) at 30 ºC.

Enzyme	Substrate	Specific activity (U/mg)		k_{cat} (s^{-1})	
		MOPS	Tris-HCl	MOPS	Tris-HCl
$CHMO_{Acineto}$	Cyclohexanone	11.4 ± 1.9	7.6 ± 1.3	11.8 ± 2.0	7.9 ± 1.3
XenB	Ketoisophorone	7.9 ± 2.2	8.4 ± 1.8	5.0 ± 1.4	5.3 ± 1.1

Data is reported as $\bar{x}$ ± 1SD (n=3).

SUPPLEMENTARY REFERENCES

1. L. C. P. Goncalves, D. Kracher, S. Milker, M. J. Fink, F. Rudroff, R. Ludwig, A. S. Bommarius and M. D. Mihovilovic, *Adv. Synth. Catal.*, 2017, **359**, 2121-2131.
2. S. Milker, M. J. Fink, N. Oberleitner, A. K. Ressmann, U. T. Bornscheuer, M. D. Mihovilovic and F. Rudroff, *ChemCatChem*, 2017, **9**, 3420-3427.
3. N. A. Donoghue, D. B. Norris and P. W. Trudgill, *Eur. J. Biochem.*, 1976, **63**, 175-192.
4. C. Peters, R. Kolzsch, M. Kadow, L. Skalden, F. Rudroff, M. D. Mihovilovic and U. T. Bornscheuer, *ChemCatChem*, 2014, **6**, 1021-1027.
5. X.-Q. Pei, M.-Y. Xu and Z.-L. Wu, *J. Molec. Catal. B Enzym.*, 2016, **123**, 91-99.
6. A. Fryszkowska, H. Toogood, M. Sakuma, J. M. Gardiner, G. M. Stephens and N. S. Scrutton, *Adv. Synth. Catal.*, 2009, **351**, 2976-2990.
7. H. Iwaki, S. Wang, S. Grosse, H. Bergeron, A. Nagahashi, J. Lertvorachon, J. Yang, Y. Konishi, Y. Hasegawa and P. C. Lau, *Appl. Environ. Microbiol.*, 2006, **72**, 2707-2720.
8. M. Y. Rios, E. Salazar and H. F. Olivo, *Green Chem.*, 2007, **9**, 459-462.
9. F. Hollmann, A. Taglieber, F. Schulz and M. T. Reetz, *Angew. Chem. Int. Ed. Engl.*, 2007, **46**, 2903-2906.
10. J. S. Zigler, Jr., J. L. Lepe-Zuniga, B. Vistica and I. Gery, *In Vitro Cell. Dev. Biol.*, 1985, **21**, 282-287.
11. D. V. Rial, P. Cernuchova, J. B. van Beilen and M. D. Mihovilovic, *J. Mol. Catal. B-Enzym.*, 2008, **50**, 61-68.
12. M. D. Mihovilovic, F. Rudroff, B. Grotzl, P. Kapitan, R. Snajdrova, J. Rydz and R. Mach, *Angew. Chem. Int. Ed. Engl.*, 2005, **44**, 3609-3613.
